# On the Inhibition Mechanism of Glutathione Transferase P1 by Piperlongumine. Insight From Theory

**DOI:** 10.3389/fchem.2018.00606

**Published:** 2018-12-10

**Authors:** Mario Prejanò, Tiziana Marino, Nino Russo

**Affiliations:** Dipartimento di Chimica e Tecnologie Chimiche, Università della Calabria, Arcavacata di Rende, Italy

**Keywords:** glutathione S-transferase, piperlongumine, hydrolysis mechanism, inhibition mechanism, MD DFT, QM, QMMM

## Abstract

Piperlongumine (PL) is an anticancer compound whose activity is related to the inhibition of human glutathione transferase of pi class (GSTP1) overexpressed in cancerous tumors and implicated in the metabolism of electrophilic compounds. In the present work, the inhibition mechanism of hydrolyzed piperlongumine (hPL) has been investigated employing QM and QM/MM levels of theory. The potential energy surfaces (PESs) underline the contributions of Tyr residue close to *G site* in the catalytic pocket of the enzyme. The proposed mechanism occurs through a one-step process represented by the nucleophilic addition of the glutathione thiol to electrophilic species giving rise to the simultaneous C-S and H-C bonds formation. Both the used methods give barrier heights (19.8 and 21.5 kcal mol^−1^ at QM/MM and QM, respectively) close to that experimentally measured for the C-S bond formations (23.8 kcal mol^−1^).

## Introduction

**Figure 1:**
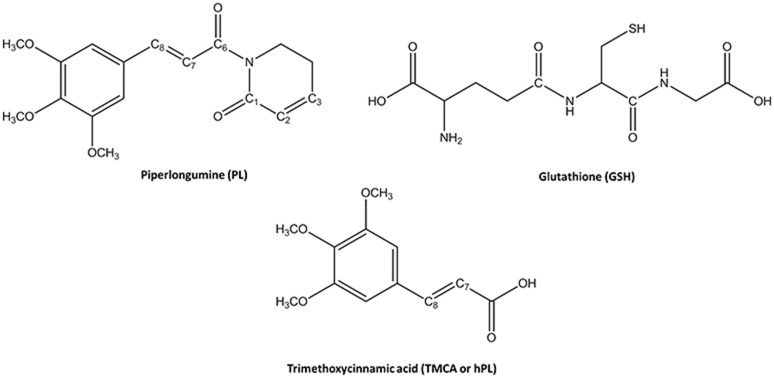
Chemical Species involved in the inhibition reaction of GSTP1.

Glutathione-S-transferases (GSTs) is a ubiquitous family of multifunctional enzymes of phase II detoxification system that conjugate reactive substrates with reduced tripeptide glutathione (GSH) in most cells, especially those in the liver and kidney (Hayes et al., 2005; Oakley, 2005; Stoddard et al., 2017). In particular, they catalyze the nucleophilic attack of the thiol group arising from cysteine residue (Cys) of the GSH on electrophilic substrates leading to formation of conjugates, that are less toxic and more water-soluble than the parent species, facilitating their elimination from cells (Broxterman et al., 1995; Townsend and Tew, 2003; Wang et al., 2017). Their role in protecting the cells from oxidative attack, in association with their overexpression in many cancer cells, makes them good candidates as cancer biomarkers (McIlwain et al., 2006; Lo and Ali-Osman, 2007). Furthermore, glutathione-S-transferases are associated with multidrug resistance of tumor cells and are involved in drug detoxification and in apoptosis control (Townsend and Tew, 2003; Mejerman et al., 2008). Mammalian cytosolic GSTs isoenzymes belong to different families or classes (alpha, mu, pi, theta, kappa, sigma, zeta, and omega) (Wilce and Parker, 1994; Armstrong, 1997; Sheehan et al., 2001) based on their molecular masses, isoelectric points and other properties. Every isoenzyme subunit contains an active site entailing a binding site for the cofactor GSH (*G-site*) and one for the electrophilic substrate (*H-site*) (Dirr et al., 1994; Wilce and Parker, 1994). In particular, the Glutathione S-transferase P1 (GSTP1) is overexpressed in different human malignancies affecting important organs as lung, colon, stomach, kidney, ovary, mouth, and testis (Green et al., 1993; Katagiri et al., 1993; Grignon et al., 1994; Okuyama et al., 1994; Zhang et al., 1994; Inoue et al., 1995; Ruiz-Gomez et al., 2000). This overexpression has been linked to acquire multidrug resistance to chemotherapeutic agents (cisplatin, chlorambucil, and ethacrynic acid) (Ban et al., 1996; Oakley et al., 1997; Mejerman et al., 2008; Karpusas et al., 2013; Pei et al., 2013; Perperopoulou et al., 2018). GSTP1 has additional role in maintaining the cellular redox state (Tew, 2007) and “nonenzymatic” antiapoptotic activity through its interaction with the *c-Jun* NH_2_-terminal kinase (JNK), a key enzyme implicated into the apoptotic cascade (Adler et al., 1999; Wang et al., 2001). For these reasons, GSTP1 is considered as a promising target for inactivation in cancer treatment and numerous researchers have spent considerable effort to propose potent inhibitors of this enzyme (Bezerra et al., 2007; Federici et al., 2009; Raj et al., 2011; Adams et al., 2012; Boskovic et al., 2013; Liao et al., 2016; Harshbarger et al., 2017; Zou et al., 2018). Among these, piperlongumine (PL) is a natural alkaloid isolated from *Piper longum* L. characterized by the presence of two α, β- unsaturated functionalities (see Figure 1) and recently has been reported as a promising anticancer molecule by targeting the stress response to ROS, inducing apoptosis (Adams et al., 2012; Boskovic et al., 2013; Liao et al., 2016; Harshbarger et al., 2017).

This molecule also represents a promising lead compound in the developing potent GSTP1 inhibitors stimulating the synthesis of a huge number of its structural analogs (Bezerra et al., 2007; Adams et al., 2012; Boskovic et al., 2013; Liao et al., 2016; Harshbarger et al., 2017; Stoddard et al., 2017). PL acts as Michael acceptor because can undergo heteroconjugate addition with the peptide-like molecules including nucleophilic thiols of cysteine residues in irreversible or reversible fashion. From stable isotope labeling (Raj et al., 2011) the anti-cancer effects of PL were related to the promotion of reactive oxygen species (ROS) and to the reduction of GSH cellular levels (Harshbarger et al., 2017). PL contains a trimethoxyphenyl head and two reactive olefins moieties (C2-C3 and C7-C8) that revealed to be essential for differentiating the cellular activity (Adams et al., 2012). The C2-C3 bond is critical for toxicity, ROS elevation and protein S-glutathionylation while C7-C8 is not necessary for these activities and is believed to enhance the toxicity (Adams et al., 2012; Harshbarger et al., 2017). This means that the two present olefins can be identified as the minimum pharmacophore of PL so that their modifications can originate analogs with different biological response (Adams et al., 2012). Furthermore, it can act as GSTP1-cosubstrate in both displacement and addition reactions. In this case, GSH bound in the G site of GSTP1 is the target of the inhibitor (Adams et al., 2012; Harshbarger et al., 2017). Recently, the high resolution X-ray crystal structure of GSTP1 (PDB code 5J41) in complex with PL and GSH proposed as the inhibition occurs without GSTP1 covalent modification by PL but, rather unexpectedly, PL results to be hydrolyzed to a trimethoxycinnamic acid (TMCA) deprived of the C2-C3 olefin (Harshbarger et al., 2017). This finding does not completely fit the behavior of PL toward other cysteine-containing peptides that react with the C2-C3 reactive bond *in vitro* conditions (Adams et al., 2012). Harshbarger et al. provided the first structural model for the interactions between PL, GSH and GSTP1 (Harshbarger et al., 2017). From this study emerged that PL acts as a prodrug. In fact, after entrance in the cell it undergoes hydrolysis giving rise to the TMCA that in turns reacts with GSH, located in the G site of GSTP1, affording the hPL:GSH conjugate as product of the addition reaction and confirming that no covalent bond formation occurs between PL and GSTP1. Although the presence in the literature of many scientific works (Bezerra et al., 2007; Federici et al., 2009; Adams et al., 2012; Boskovic et al., 2013; Peng et al., 2015; Liao et al., 2016; Harshbarger et al., 2017; Zou et al., 2018) on the piperlongumine selective inhibition of tumor growth in different types of cancers, the molecular mechanism involved in PL mediated cancer cell death remains still poorly understood. With the aim to contribute to a better knowledge, at atomistic level, of the inhibition mechanism of GSH by the hydrolyzed product of PL into the GSTP1 enzyme, a theoretical investigation in the framework of density functional theory (DFT) was undertaken. In addition, a MD simulation of initial enzyme-inhibitor (EI) complex has been also performed.

## Methodology

### Active Site

**Figure 2:**
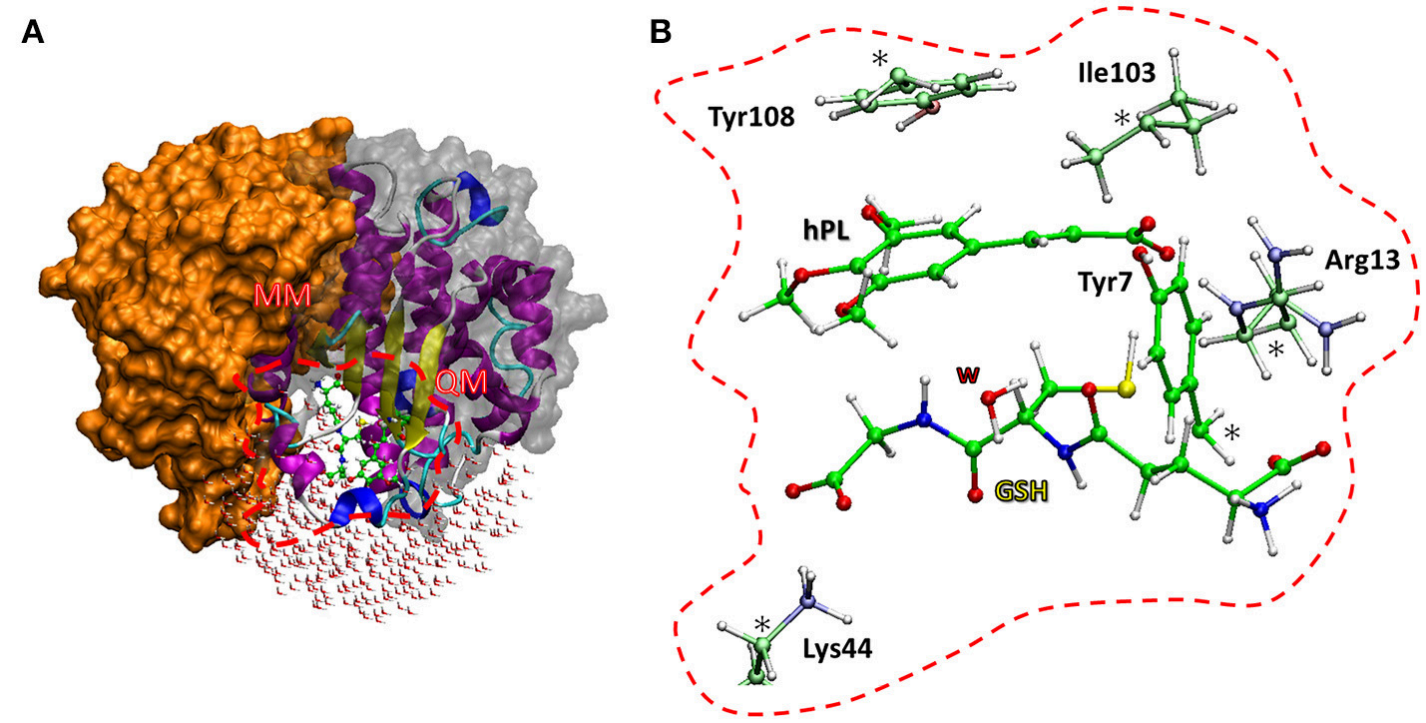
The two models used. The QM/MM model **(A)** includes the whole protein with the water molecules, the red box defines the QM region used in both cluster **(B)** and QM/MM calculations. The amino acid residues of the QM portion are shown with ball and sticks representation.

The enzyme structure includes two identical homodimers, with a total mass of 48 kDa. The active sites are located in the interfaces between the two domains. Each active site in turn consists of two sub-sites: the *G site*, where GSH is located, in proximity to the outer side of protein surface and in direct contact with solvent molecules, and the *H site*, where the electrophilic inhibitor can be accommodated (Harshbarger et al., 2017). The interactions between GSH and hPL with different residues of the cavity of both sites were treated at quantum mechanical level in both QM and QM/MM calculations. In particular, the QM region includes: the Arg13 which is engaged in hydrogen bonds with the N-terminal portion of GSH and the carboxyl group of inhibitor, the Lys44 which is anchored to C-terminal part of GSH, the Tyr7 with its OH moiety oriented toward the S atom of GSH-cysteine in such a manner to establish H bond between them, and Tyr108 which is involved in π-π interaction with inhibitor aromatic ring. Finally, the QM portion, contains also the Ile104 since its crucial role in correctly orienting hPL (in *H site*) during the conjugation phase with GSH (Harshbarger et al., 2017). Due to the closeness of active site to the protein surface, several water molecules present in the catalytic cavity were considered in the QM/MM model. Starting from the available crystallographic structure of GSTP1 by *Homo Sapiens* (Harshbarger et al., 2017; PDB code 5J41, 1.19 Å resolution), the preparation of the models (see Figure 2) is described by the following procedure.

### MD Calculations

As first step of the work it was necessary to perform the C8_hPL_-S_GSH_ bond cleavage and then to relax the enzyme:GSH:inhibitor supramolecular system at the molecular mechanics (MM) level of theory before starting the MD simulation because the used X-ray structure was related to the final product of the inhibition process. Furthermore, the presence of the inhibitor molecule of non-protein nature implied its optimization at HF/6-31G(d) level of theory in order to derive the parameters by Antechamber tool, as implemented in AMBER 16 package (AMBER 16, 2016). Intramolecular Lennard-Jones parameters and atomic charges were obtained using, respectively, General Amber Force Field (GAFF) (Wang et al., 2004) and Restrained Electrostatic potential (RESP) method (Bayly et al., 1993). The obtained parameters of hPL are collected in Table S1.

The amber ff14SB (Maier et al., 2015) force field was applied using the Xleap module and hydrogen atoms were added to the whole system. The protonation state of each amino acid has been assigned using the H++ web server (Gordon et al., 2005; Myers et al., 2006; Anandakrishnan et al., 2012; H++ vesion 3.2, 2016). A rectangular box (85 × 70 × 80Å) was filled with TIP3P (Jorgensen et al., 1983) water molecules within 12.0 Å from the surface of the enzyme. The classical MD simulation was applied for 100 ps in NVT ensemble with a progressive heating phase, from 0 to 310 K. A final MD production of 20 ns was obtained in NPT ensemble (1 bar and 310 K). During the simulations, a cutoff radius for non-bonded interactions was fixed at 12 Å and Particle Mesh Ewald summation method (PME) (Ewald, 1921) and SHAKE algorithm (Ryckaert et al., 1977) were employed to constrain the motion in H-including bonds, in order to use a 2 fs integration step The root-mean-square deviation (RMSD) analysis of the whole protein and the *H* and *G* active sites residues was performed to verify the stability of the system during the MD simulation (Figure S1). To better examine the conformational behavior of the inhibitor-protein system, a MD simulation has been also performed on the alone enzyme. The obtained root-mean-square fluctuation (RMSF) is shown in Figure S2. Furthermore, in order to verify conformational homogeneity for inhibitor binding modes in to the catalytic pocket, 20 structures were selected along MD simulation (Figure S3). Clustering results confirmed that the last frame, obtained at 20 ns, is a good representative configuration as to be adopted as starting configuration for creating QM cluster and QM/MM model.

### QM Cluster and QMMM Models

The amino acids considered in the QM region (Tyr7, Arg13, Tyr103, Ile104, Lys44) were truncated as depicted in Figure 2. The missing hydrogens were added manually and one water molecule (lying at 3.601 Å from the GSH) was explicitly considered, being implicated in direct interaction with nucleophilic agent while the other waters are located away than 4 Å. The C atoms labeled with “^*^” were kept fixed during geometry optimizations, applying the locking scheme, to prevent artificial movements (Siegbahn and Himo, 2011; Piazzetta et al., 2015; Himo, 2017). The QM cluster model was found to be adequate in the elucidation of the catalytic mechanism followed by other enzymes (Amata et al., 2011; Lan and Chen, 2016; Prejanò et al., 2017). The obtained model consists of 136 atoms with a total charge equal to zero.

The QM/MM model was obtained applying the two layers ONIOM formalism (Svensson et al., 1996) as implemented in Gaussian09 code (Frisch et al., 2013), maintaining the same atoms mentioned in QM cluster model setup. The entire enzyme and a number of water molecules within 5 Å from the catalytic site were considered (Figure 2). During the optimization, all the water molecules and residues out of 18 Å sphere from the active site were kept frozen, applying the standard procedure for single conformation PES studies (Sousa et al., 2017). The final model contains 7811 atoms.

### Technical Details

Gaussian 09 (Frisch et al., 2013) software package was used to perform calculations using B3LYP (Lee et al., 1988; Becke, 1993) hybrid functional in QM region of both used models. For S, N, H, O and C atoms, 6-31+G(d,p) basis set was used during the optimizations. Linear transit scans were performed, in order to detect stationary points along reaction coordinates. To confirm the nature of intermediates or transition states, frequencies calculation was performed at the same level of theory, for each stationary point intercepted along potential energy surface (PES). To obtain more accurate electronic energies single point calculations with 6-311+G(2d,2p) larger basis set were performed. The final energy profiles include the zero point energy (ZPE) and dispersion corrections (evaluated using the DFT-D3 procedure; Grimme et al., 2011) and solvation energy.

The electrostatic embedding as implemented in Gaussian 09 was employed to evaluate the Coulomb interactions between MM and QM regions in all calculations (Vreven et al., 2006). For the QM cluster calculations, single point calculations adopting the SCRF-SMD solvation model with a dielectric constant ε = 4, simulating the enzyme environment, was used (Marenich et al., 2009). The same level of theory was adopted during the optimizations of species involved in hydrolysis of PL, considering the dielectric constant of 78.0, as successfully proposed in other studies (Ritacco et al., 2015; Marino et al., 2016). NBO (NBO version 3.1, 2001) and non-covalent interaction (NCI) (NCIPLOT, version 3.0, 2011) analyses were performed on all the stationary points of the investigated PESs at both QM and QM/MM levels.

As far as the proton affinity calculations for establishing the oxygen carbonyl to be considered in the hydrolysis mechanism at acidic conditions, the proton affinities as binding energies (BE) have been estimated as indicated by the following expression:

$$\begin{array}{l} \text{BE}=-\left(\Delta {\text{H}}_{\text{hPL}-\text{H}+}-\Delta {\text{H}}_{\text{hPL}}\right) \end{array}$$

The BE is calculated as the difference between the enthalpy of the protonated system and that of the neutral one. In the calculations, the H^+^ contribution does not appear since we evaluated the energetic difference, therefore the obtained binding energies represent the energy involved in the formation of the protonated systems.

## Results and Discussion

### Hydrolysis of Piperlongumine

**Figure 3:**
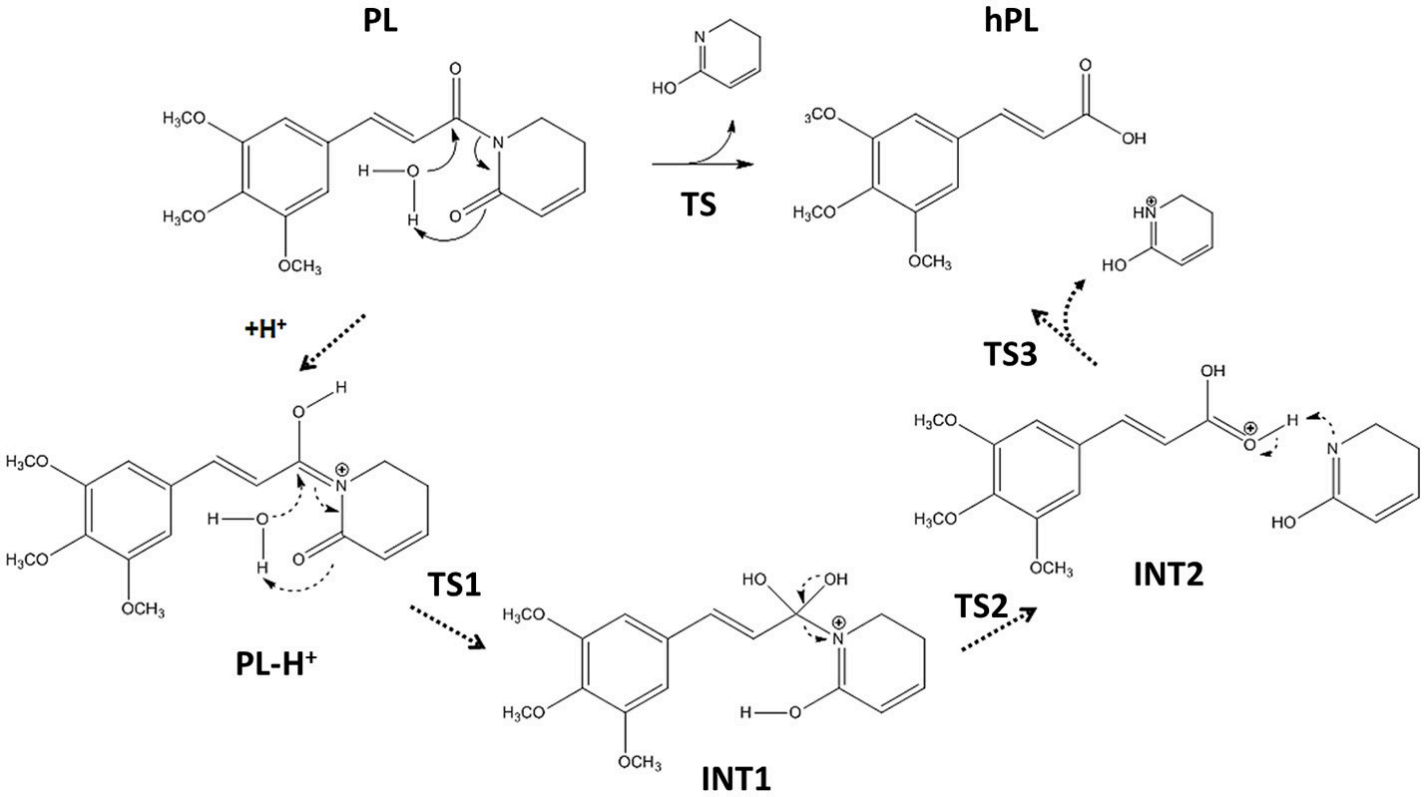
Hydrolysis mechanism of PL in neutral (bold line) and acidic conditions (dashed line).

Following the recent experimental indications that demonstrate as the PL suffers hydrolysis out of the enzyme pocket, (Harshbarger et al., 2017) we firstly study this process in aqueous media. The considered reaction mechanism is illustrated in Figure 3. As from the experimental evidence, (Harshbarger et al., 2017) we considered the hydrolysis mechanism of PL to occur on the oxygen of the carbonyl (C6) functionality next to C7-C8 olefin, under both neutral and acidic conditions to take into account the different intracellular pH conditions, since the acid pH values are observed in cancer cells (Townsend and Tew, 2003; Wang et al., 2017).

On the contrary, our computed BE shows that the carbonyl moiety next to C2-C3 olefin has minor proton affinity (about 4 kcal mol^−1^) with respect to that next to C7-C8 one, indicating as under the same conditions the favored protonation site is the oxygen of C6.

**Figure 4:**
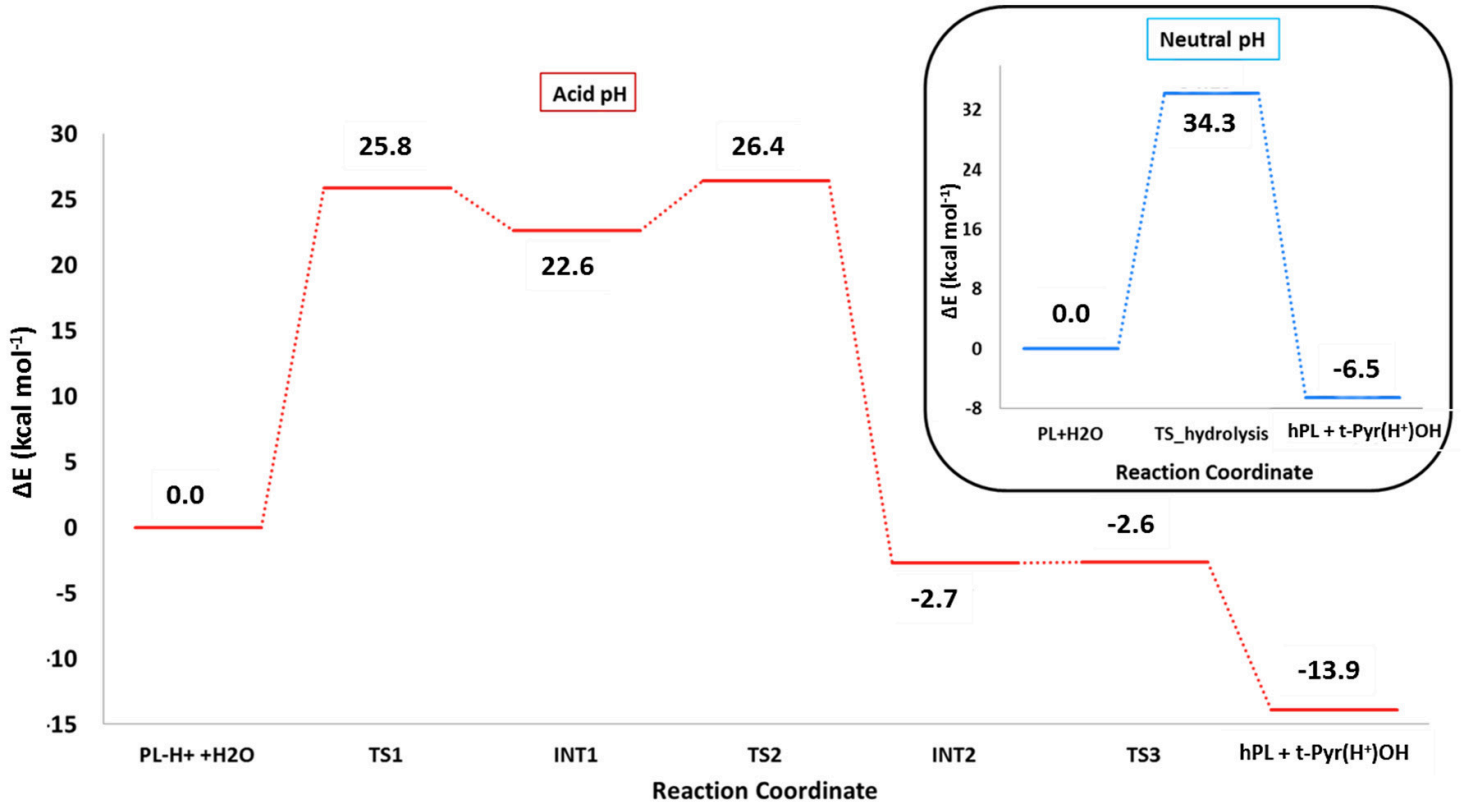
B3LYP-D3/6-311+G(2d,2p)//B3LYP/6-31+G(d,p) (SMD,ε = 78) energy profiles of PL hydrolysis in neutral conditions (blue line) and acid conditions (red line). The final energies contain ZPE and D3 corrections.

The optimized geometries of the stationary points are reported in (Figures S4, S5), while the calculated energy profiles are depicted in Figure 4. As shown from Figure 3, we propose at acid pH a mechanism occurring in a multistep process contrary to that at neutral conditions occurring in only one step. In both cases the product is the hPL, while the leaving group is 1,2,5,6-tetra-hydro-pyridin-2-ol (t-PyrOH) neutral and protonated, respectively. For clarity, in the text the remaining double bond in the hPL upon hydrolysis will retain the same numeration than in PL, (C7-C8). The processes are exothermic although at pH acid the exoergonicity is more pronounced (Figure 4). From our results, the acidic hydrolysis is strongly favored as suggested by lower activation barriers (by about 10 kcal mol^−1^) than that found at neutral pH (see Figure 4). The calculated barrier in acidic environment well-agrees with those characterizing other anticancer molecules acting as prodrug (Alberto and Russo, 2011; Ritacco et al., 2015; Marino et al., 2016). Once the hydrolyzed product is formed, the process of GSH-conjugation favored by GSTP1 starts through the attack to the C7-C8 double bond.

### GSTP1 Inhibition

**Figure 5:**
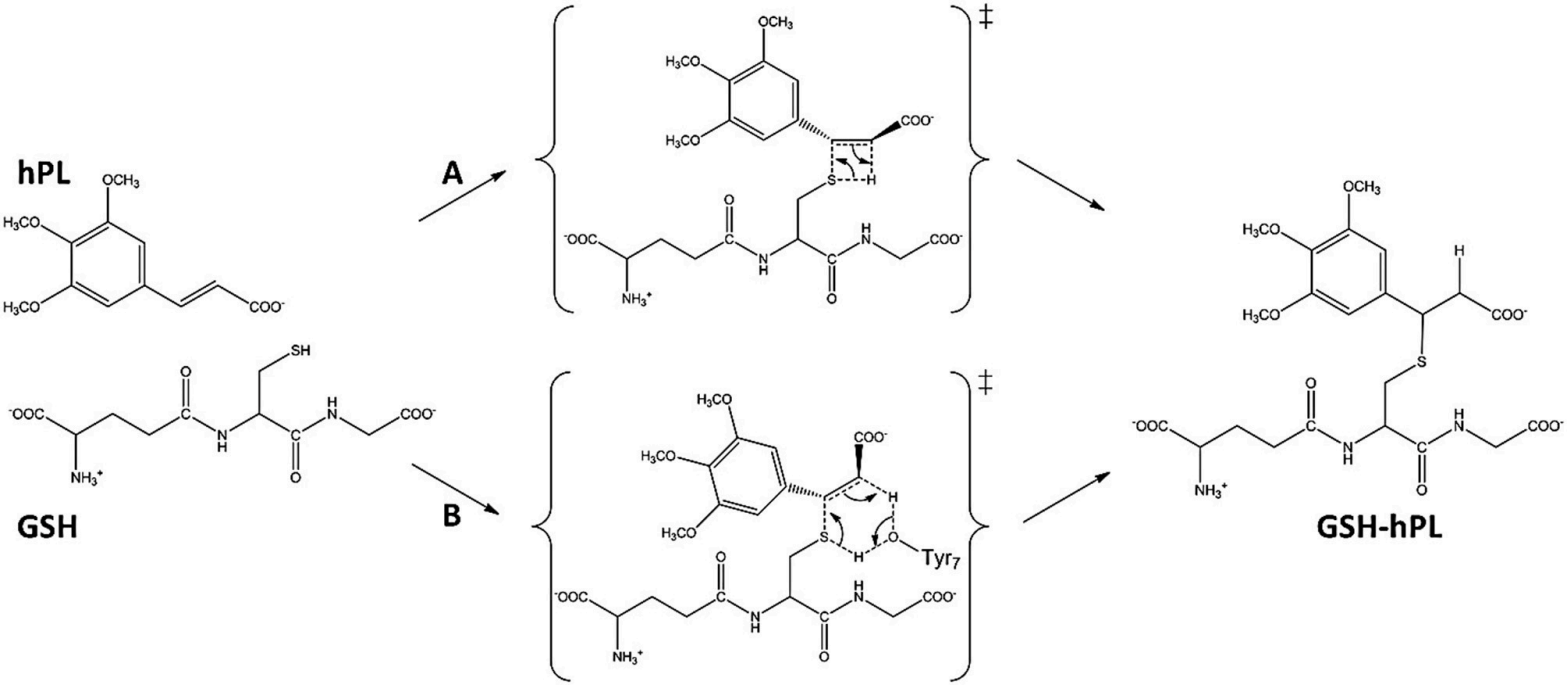
Proposed mechanisms for GSTP1 inhibition by hPL.

**Figure 6:**
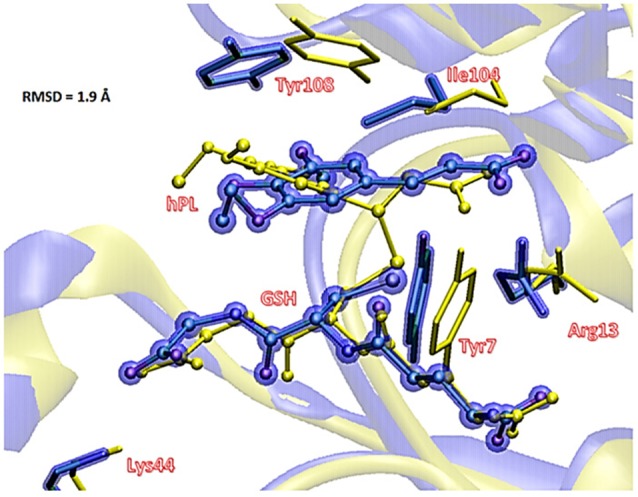
Superposition of the ternary adduct (EI) from MD (violet) with the X-ray structure (yellow) related to the P S-conjugate product.

To underline the role of GSTP1 during the inhibition process by hPL, we have considered, at both QM and QM/MM levels, two different reaction mechanisms (**A**, and **B**) as presented in Figure 5. In particular: (**A**) describes the nucleophile addition to the double bond of inhibitor by -SH group of GSH without involving any amino acid residue while path (**B**) takes into account the participation of the Tyr7 residue in the formation of the covalent adduct. In all the cases, the inhibition reaction occurs in a one-step process by the Michael addition of the thiol from GSH at the C7-C8 olefin of hPL. In all the considered mechanisms, the starting species is the ternary enzyme-hydrolyzed inhibitor-GSH complex (**EI**) obtained after the geometry optimization of the frame isolated by the previous MD cycle at 20 ns. From Figure 6, that illustrates the superposition of the crystallographic structure with the last MD snapshot, it is possible to note that hPL interacts with the binding cleft of the *H site* and no water molecules are close to the reaction center, in agreement with the hydrophobic nature of site (Tyr7, Tyr108, and Ile104 residues). As expected, the crystallographic pose (obtained with the reaction product) deviates in this moiety (see Figure 6). At the contrary, the GSH region is well-superimposed confirming that this molecule is well-positioned with a correct orientation of the thiol.

**Figure 7:**
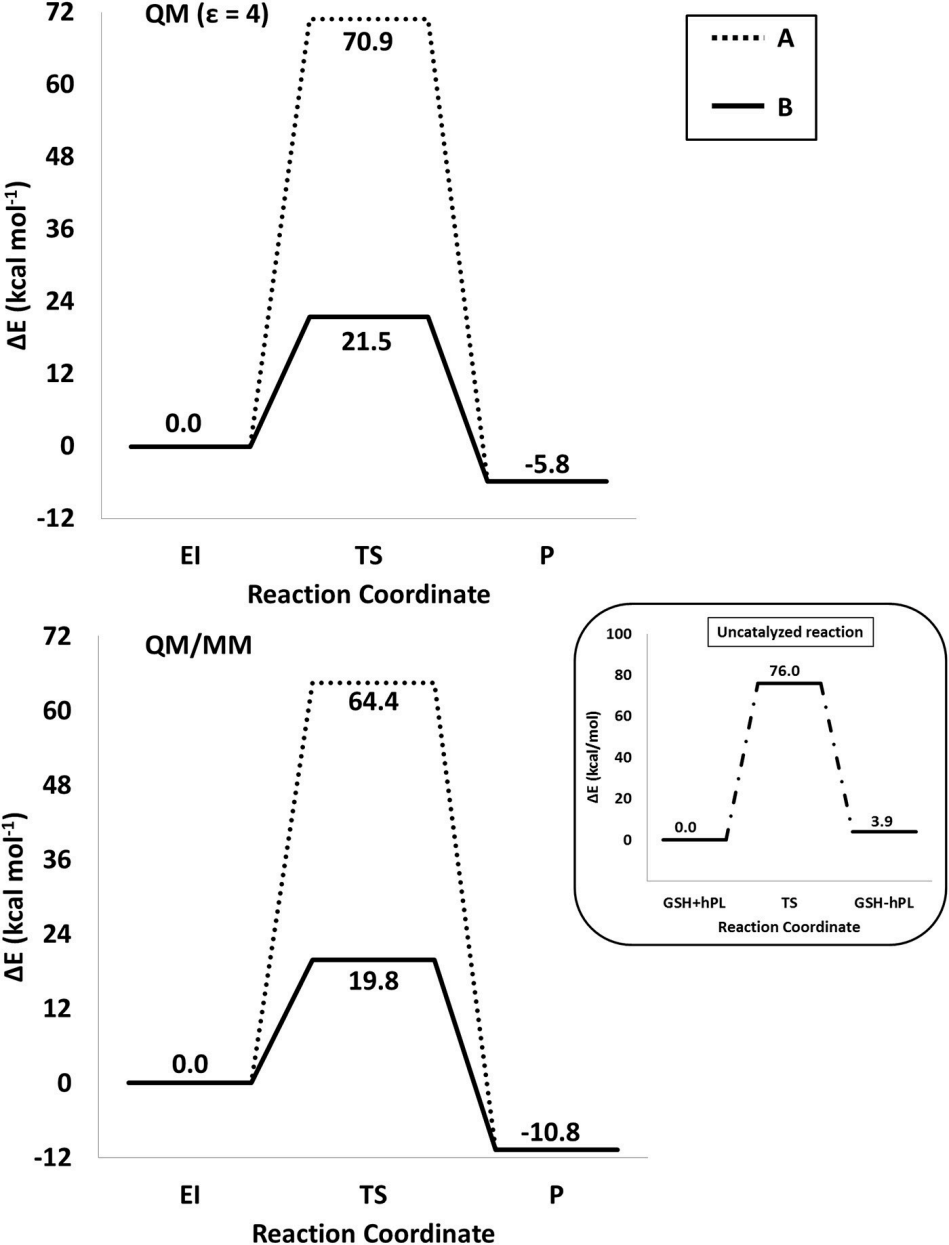
/B3LYP-D3/6-311+G(2d,2p)//B3LYP/6-31+G(d,p) (SMD,ε = 4) (top) and B3LYP-D3/6-311+G(2d,2p):ff99SB//B3LYP/6-31+G(d,p):ff99SB (bottom) energy profiles of GSTP1 inhibition process by hPL for (A,B) mechanisms. In the black window are depicted the energy profile related to the reaction unassisted by the enzyme. The final energies contain ZPE and D3 corrections.

**Figure 8:**
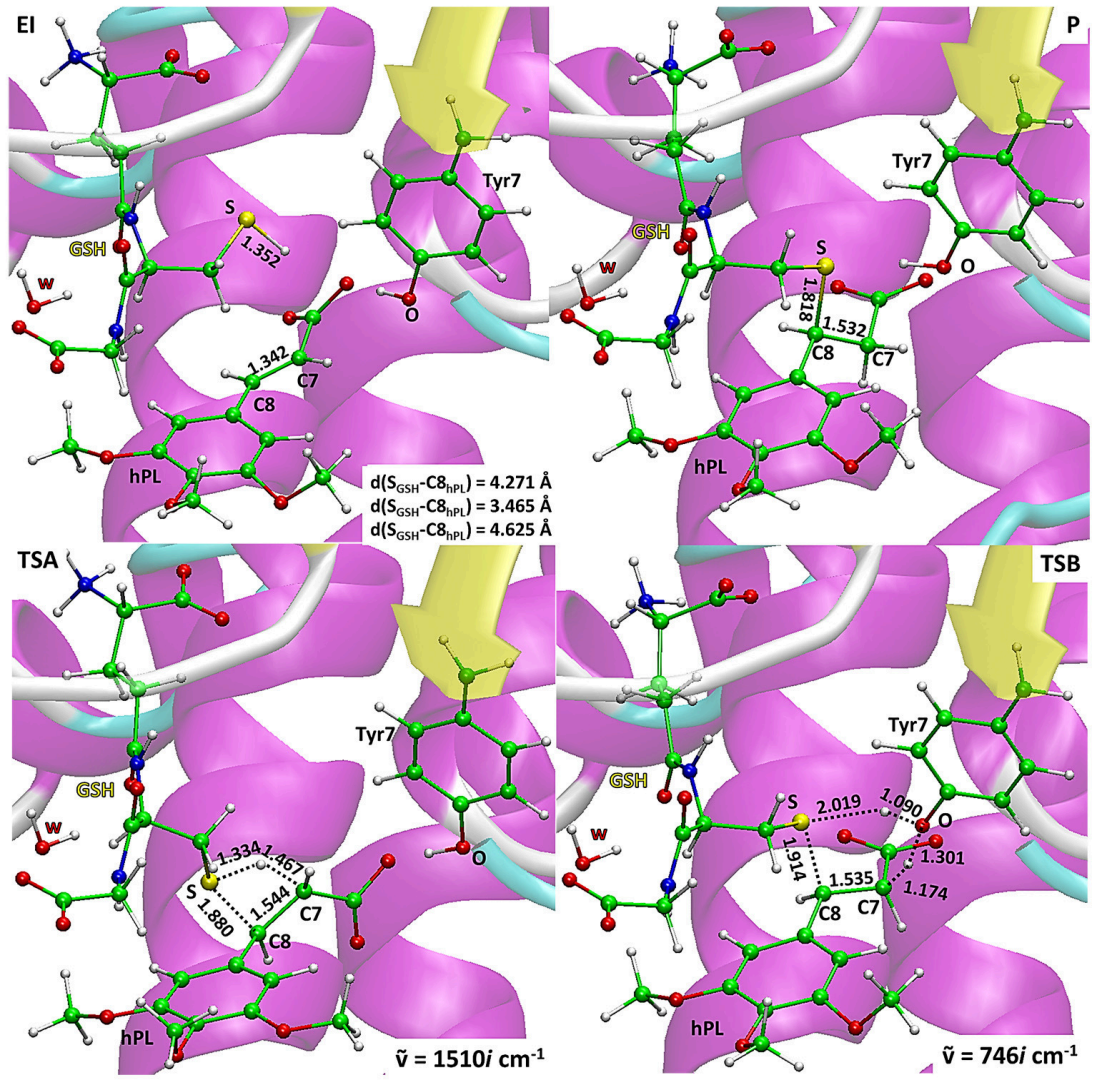
B3LYP/6-31+G(d,p):ff99SB optimized geometries of EI, P, TSA, TSB. For the transition states the imaginary frequencies are reported.

The energy profiles obtained employing QM and QM/MM tools for the two considered mechanisms, are reported in Figure 7. The reported QMMM energy values do not include the entropic contribution. In order to quantify this the Grimme procedure has been employed (Grimme, 2012). Results (see Table S2) evidence that the TΔS terms slightly affect the previously obtained energy values. QM/MM structures of the initial complex (**EI**), the final S-conjugated product (**P**) and that of the transition states are reported in Figure 8. All the QM optimized geometries are given in (Figure S6).

**Figure 9:**
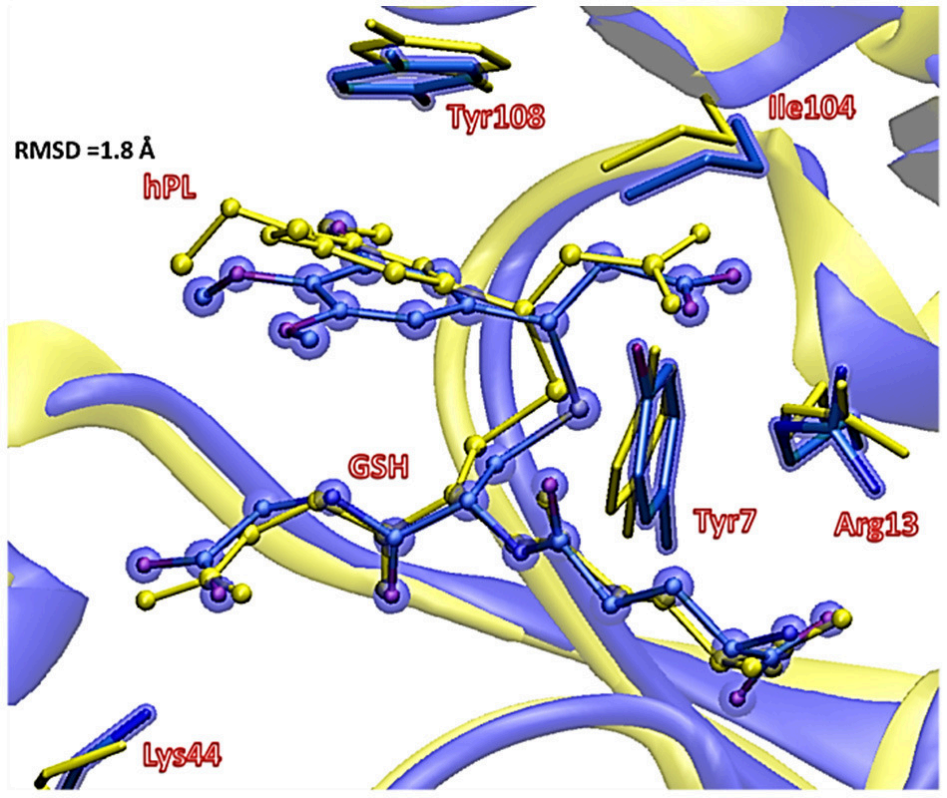
Superposition of the ONIOM optimized structure of the final hPL:GSH conjugated product (violet) with its corresponding crystallographic structure characterized (yellow).

In **EI** the carboxylate moiety of the hPL is oriented in such a way to establish hydrogen bonds with Tyr7 (1.599 Å) and SH group of GSH (2.138 Å). Furthermore, van der Waals and hydrophobic interactions, such as those between the inhibitor and the Ile104 and Arg14 residues (see Figure 8) contribute to optimally accommodate the inhibitor into the H binding site. In fact, now the key reacting atoms, C8 of the hPL and SH nucleophile species, are placed in suitable way (at 4.271 Å) to allow the deactivation reaction. In path **A** the intercepted transition state (**TSA**) represents a four-centered structure where the sulfur addition to the C8 (1.880 Å) and the C7-H bond formation (1.467 Å) simultaneously occur. The corresponding frequency analysis confirms a first-order saddle point with an imaginary frequency (1510*i* cm^−1^) which corresponds to a vibrational mode involving a strong C7–H coupling and a relatively weaker C8–S one. The C8-S bond is already established and the forming C-H one can be evinced by the elongation of the C7-C8 bond (1.544 Å). This barrier results to be 64.4 kcal mol^−1^ at QM/MM level and 70.9 kcal mol^−1^ at QM one. Both values are very close to that computed for the reaction unassisted by the catalyst (76.0 kcal mol^−1^, see Figure 7). The resulting product (**P**), shown in Figure 8, evidences that the C-S bond is formed (1.818 Å) and the C8-C7 is elongated (1.532 Å) confirming the occurred sp^3^ hybridization of the two involved carbon atoms. The exothermicity is evaluated to be 10.8 kcal mol^−1^ (5.8 kcal mol^−1^ in the QM cluster). The mechanism **B** (Figure 5) involves the participation of the Tyr7 residue. In Figure 8 is reported the optimized structure of the **TSB** connecting the **EI** and the covalent final complex (**P**). The nucleophilic attack to C8 occurs by GSH-thiolate (1.914 Å) since the hydrogen of the S-H_GSH_ group (2.019 Å) has been delivered to oxygen (O_Tyr_) of the side chain of Tyr7 (1.090 Å). In fact, the OH group of Tyr7, oriented via hydrogen bonding to carboxylate moiety of the inhibitor (1.599 Å), in the TS becomes 1.310 Å and points toward C7 atom for delivering its hydrogen atom (O-H and H-C7 distances are found to be 1.310 and 1.174 Å, respectively) while the C7-C8 bond is elongated (1.535 Å) (see Figure 8). The TS located along the mechanism **B** lies at 21.5 kcal mol^−1^ (QM) and 19.8 kcal mol^−1^ (QM/MM) above the **EI**. Both values are very close to the available experimental one (23.8 kcal mol^−1^) concerning the C-S bond formation (Huskey et al., 1991). The superposition of our optimized glutathionil-conjugated product **P** with the corresponding crystallographic structure (Harshbarger et al., 2017; see Figure 9) reveals a good RMSD value in both GSH and *H site* regions. The exothermicity 10.8 kcal mol^−1^ means that the reverse reaction can be accessible but much slower also for the high barrier required in the reverse process **P EI** (30.6 kcal mol^−1^). **TSB** evidence as the formation of the S-C8 bond is strictly related to the deprotonation of the SH moiety of GSH at the expense of the Tyr7 acting as proton shuttle with a consequent reduction of barrier (19.8 kcal mol^−1^).

**Figure 10:**
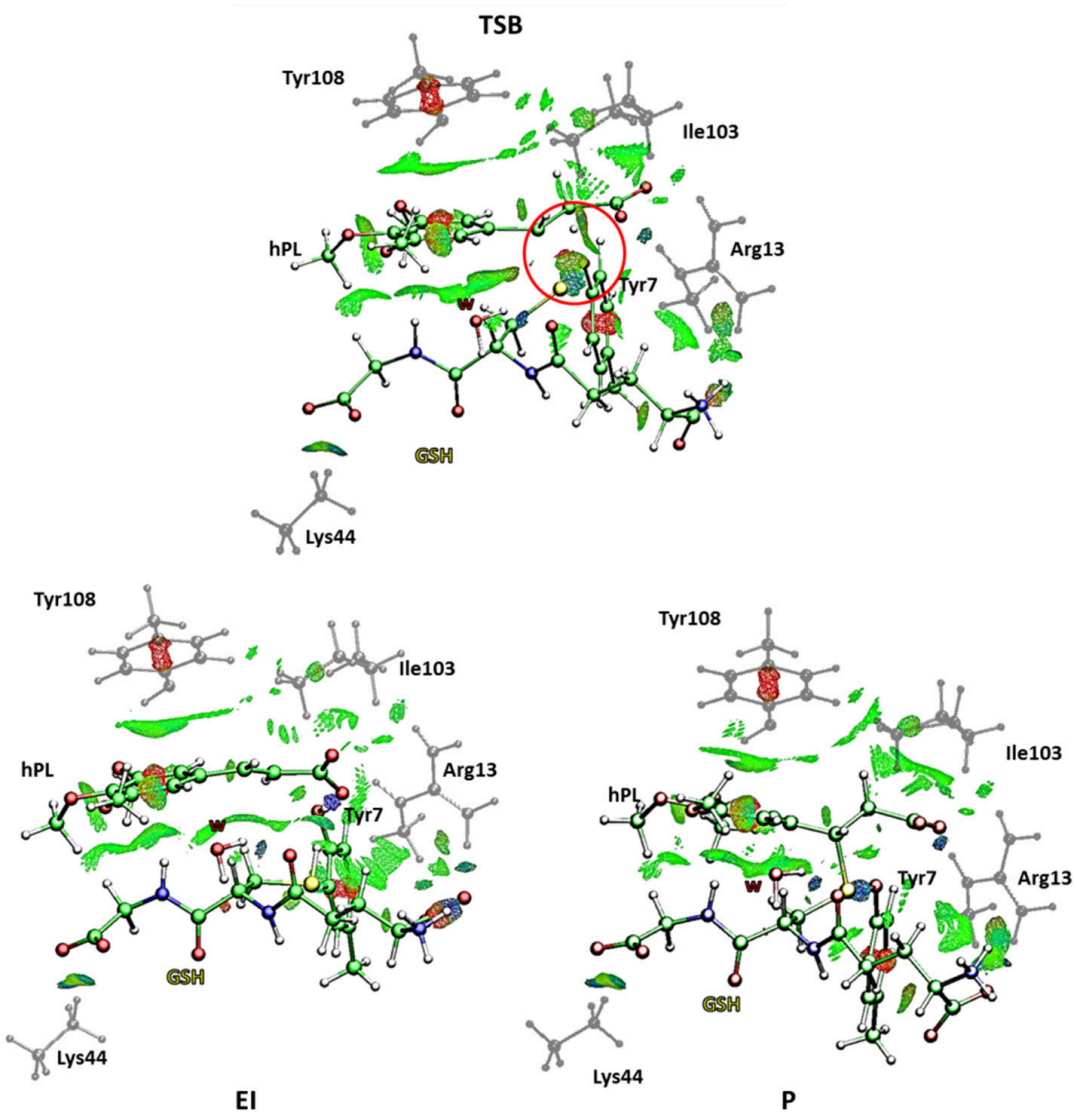
Nonbonding interaction plot calculated for the stationary points at B3LYP-D3/6-311+G(2d,2p)//B3LYP/6-31+G(d,p) level for the B mechanism. The red circle defines the portion where the bonds breaking and formation occur.

This is in agreement with previous works on other GST enzymes (Zheng and Ornstein, 1997; Angelucci et al., 2005; Dourado et al., 2008) revealing the importance of the acidic properties of a Tyr during the catalysis of glutathione-S-Transferase. Furthermore, our findings corroborate the hypothesis advanced by the previous structural analysis (Harshbarger et al., 2017) revealing as no covalent bond formation between hPL and GSPT1 was observed and that PL acts as prodrug. With the aim to evaluate the nature of the interactions present inside the catalytic pocket during the process, in Figure 10 we have reported the density of isosurfaces arising from NCI analysis, indicating the different contributions of the residues retained in the QM region.

In every characterized stationary point, it can be noted the salt bridges occurring between the side-chains of Lys44 and Arg13 with the carboxyl moiety of carboxyl- and amino-terminal of GSH (blue region indicates strong attractive interactions while the red isosurfaces account for the repulsive interactions related to the center of π systems of Tyr7 and Tyr108 and the inhibitor molecule, as usually for aromatic systems strong non-bonded overlap is indicated (Johnson et al., 2010). Further information arises from the green regions indicative of the van der Waals forces characterizing the cavity containing the inhibitor molecule and identified by the hydrophobic residues Tyr108 and Ile104. It is interesting to underline as the interaction involving the Ile104 becomes more intense as the reaction proceeds. At the contrary no relevant contributions arise from the NBO analysis (see Table S3) except for a little bit increased nucleophile nature of the sulfur atom of GSH in the enzyme and a decreased negative charge on the C7-C8 bond of the hPL species with the respect to the corresponding values obtained for the process unassisted by enzyme. In the **TSB** species, a more attractive interaction appears in proximity of the region interested in the chemical events (circled in red in Figure 10) symptomatic of the occurring S-C8 bond formation. Furthermore, the interatomic distances, during the mechanism, between the residues of the QM region and the GSH and hPL species, reported in Table S4, highlight how no significant change occurs in the catalytic pocket.

## Conclusion

This study focuses on the inhibition mechanism of the glutathione-S-transferase Pi 1 by the hydrolyzed product of piperlongumine. We propose the mechanism following the most recent experimental evidences taking into account in particular the role of Tyr7 on the complex formation between the glutathione and the inhibitor inside the catalytic pocket of enzyme.

The hydrolysis of PL for giving hPL has been considered in neutral and acid conditions. The last **one** provided the better energetic path.

The agreement between cluster QM and the more computational demanding hybrid QM/MM methods is quite good. Structural and energetic computed properties are in line with the available experimental data.

The lowest energy reaction mechanism for reaction of hPL with GSH corresponds to that in which the Tyr7 residue is involved in the inhibition reaction deprotonating the GSH and donates the proton, in a concerted fashion, to the C7 substrate atom. The computed barrier heights result 19.8 and 21.5 kcal mol^−1^ in both QM/MM and QM models, respectively. Both computations clearly indicate the same reaction mechanism by TSB as the preferred one with difference in the barrier eight is of only 1.7 kcal mol^−1^and propose an exergonic reaction.

We hope that the obtained new insights on the reaction mechanism of human GSTP1 inhibition with natural piperlongumine substrate can be useful in the design of new selective and more potent inhibitors.

## Author Contributions

MP, TM, and NR have analyzed the results, edit, and reviewed equally the manuscript. MP, TM, and NR approved it for publications.

### Conflict of Interest Statement

The authors declare that the research was conducted in the absence of any commercial or financial relationships that could be construed as a potential conflict of interest. The reviewer KS and handling Editor declared their shared affiliation.
