# Supplementary material for: On the Inhibition Mechanism of Glutathione Transferase P1 by Piperlongumine. Insight From Theory
Source: Front Chem. 2018 Dec 10;6:606. doi: 10.3389/fchem.2018.00606 (PMC6296316; doi:10.3389/fchem.2018.00606)
Supplement: Supplementary file 1 [file Data_Sheet_1.PDF]

Supplementary Material

On the inhibition mechanism of glutathione transferase P1 by piperlongumine. Insight from theory

Mario Prejanò, Tiziana Marino[*], Nino Russo

*** Correspondence:** Tiziana Marino: tiziana.marino65@unical.it

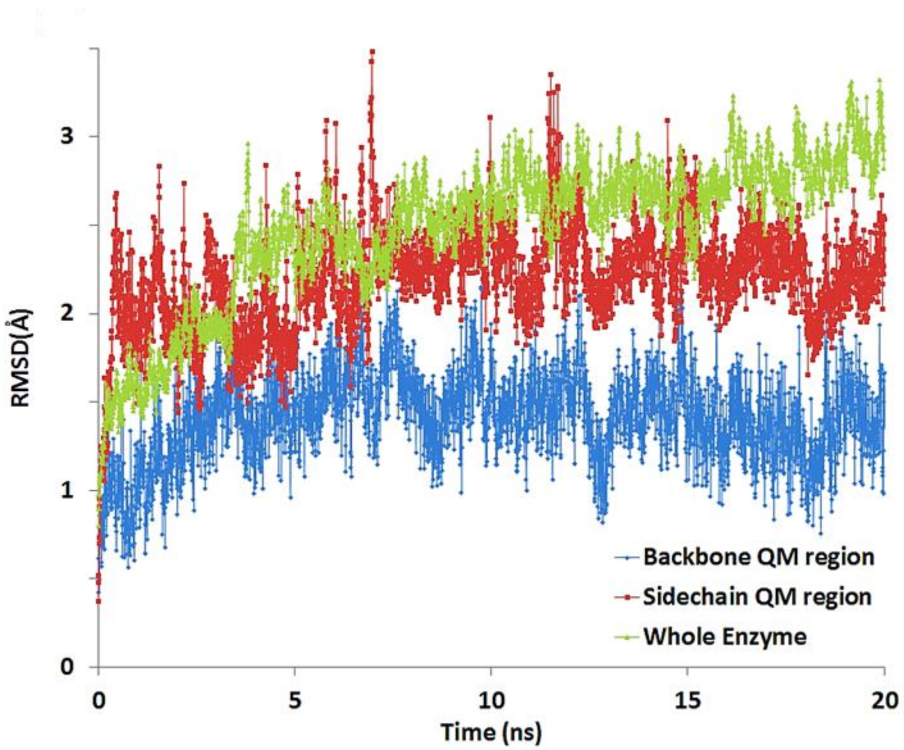

Figure S1. Root mean square deviation (RMSD) of entire enzyme (green line), of sidechain (red line) and backbone (blue line) of amino acids of *H* and *G* sites.

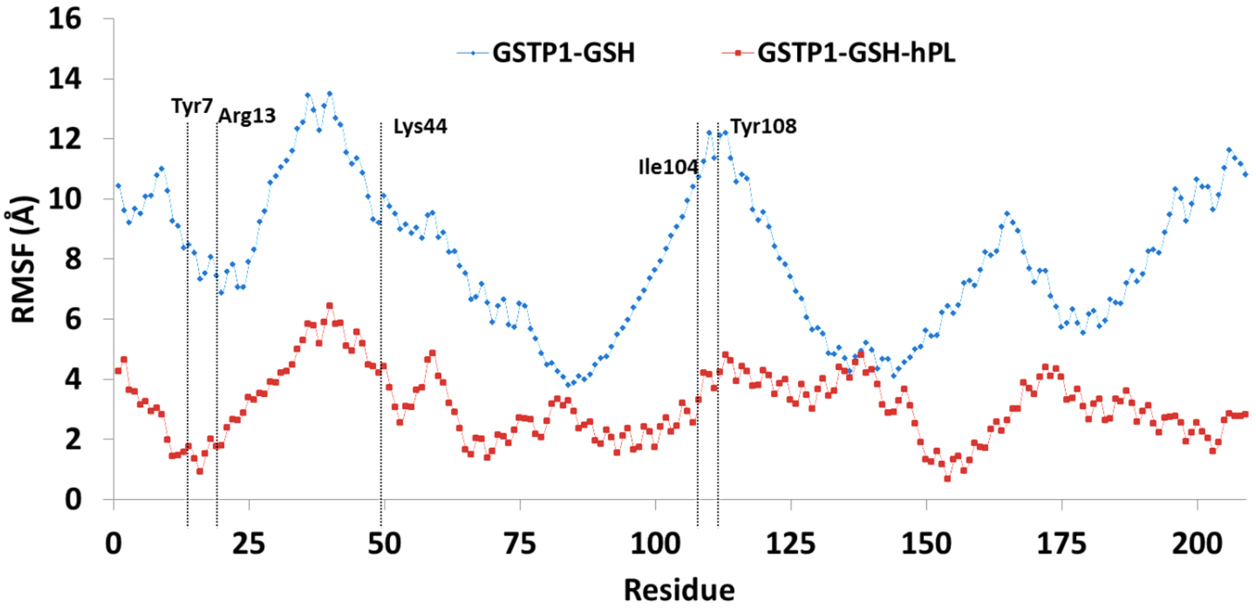

Figure S2. Root mean square fluctuation (RMSF) of residues for alone enzyme (blue line) and enzyme-inhibitor (red line).

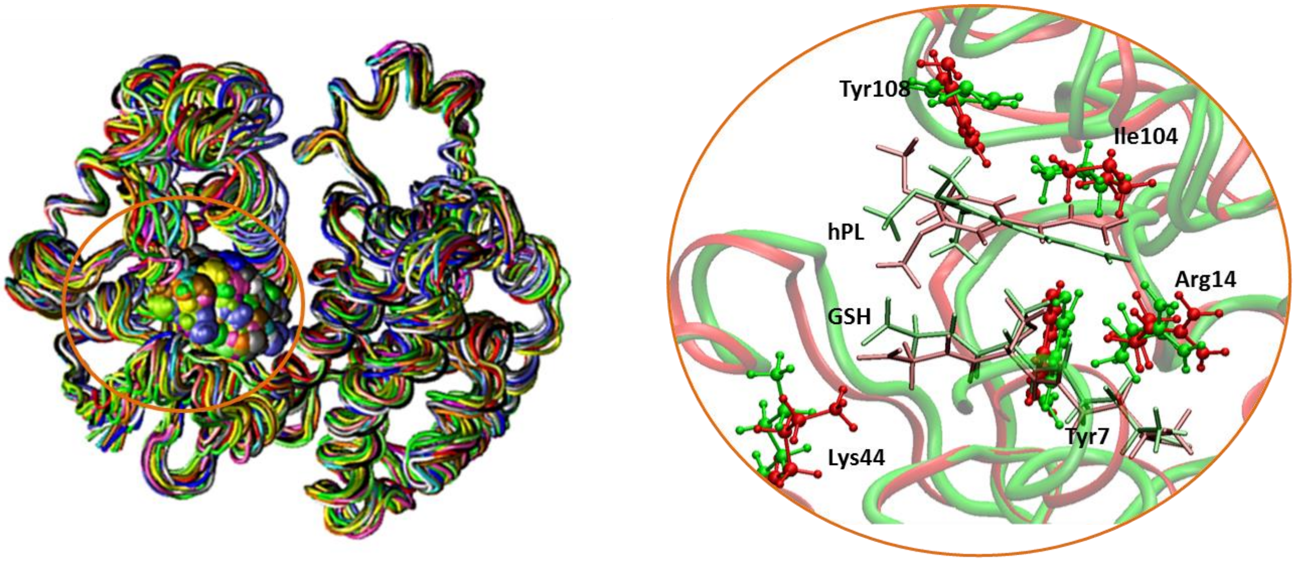

Figure S3. On the left, superposition between 20 representative structures after 20 ns of the hPL:GSH:GSTP1 system, arising by cluster analysis. On the right, the superposition of the two most representative configurations, chosen for QM portion. The configuration adopted corresponds to the one highlighted in green, presenting the lowest value of RMSD (1.9 Å).

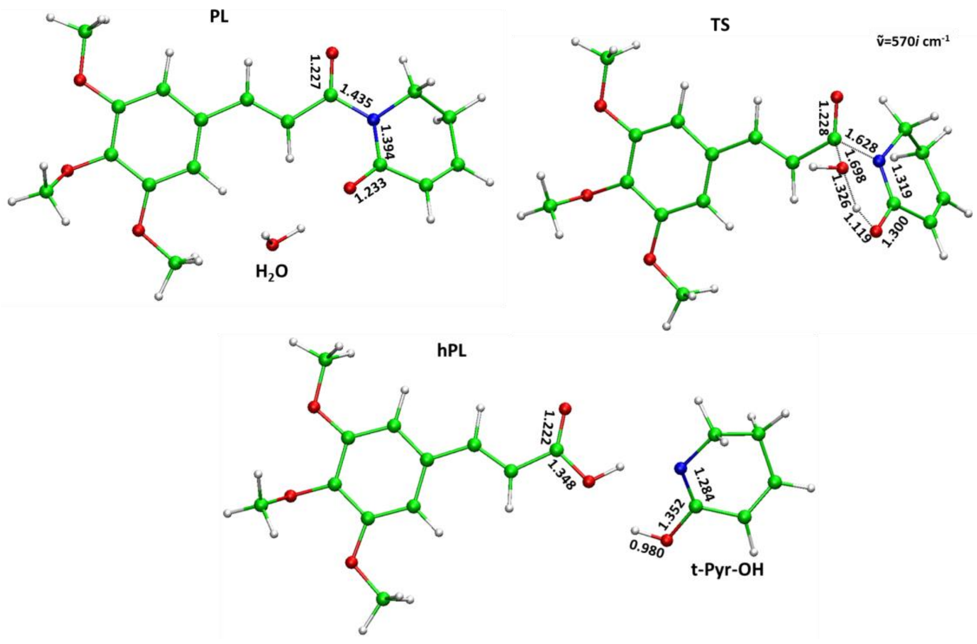

Figure S4. Optimized geometries at B3LYP/6-31+G(d,p) level of theory for hydrolysis of PL in neutral conditions.

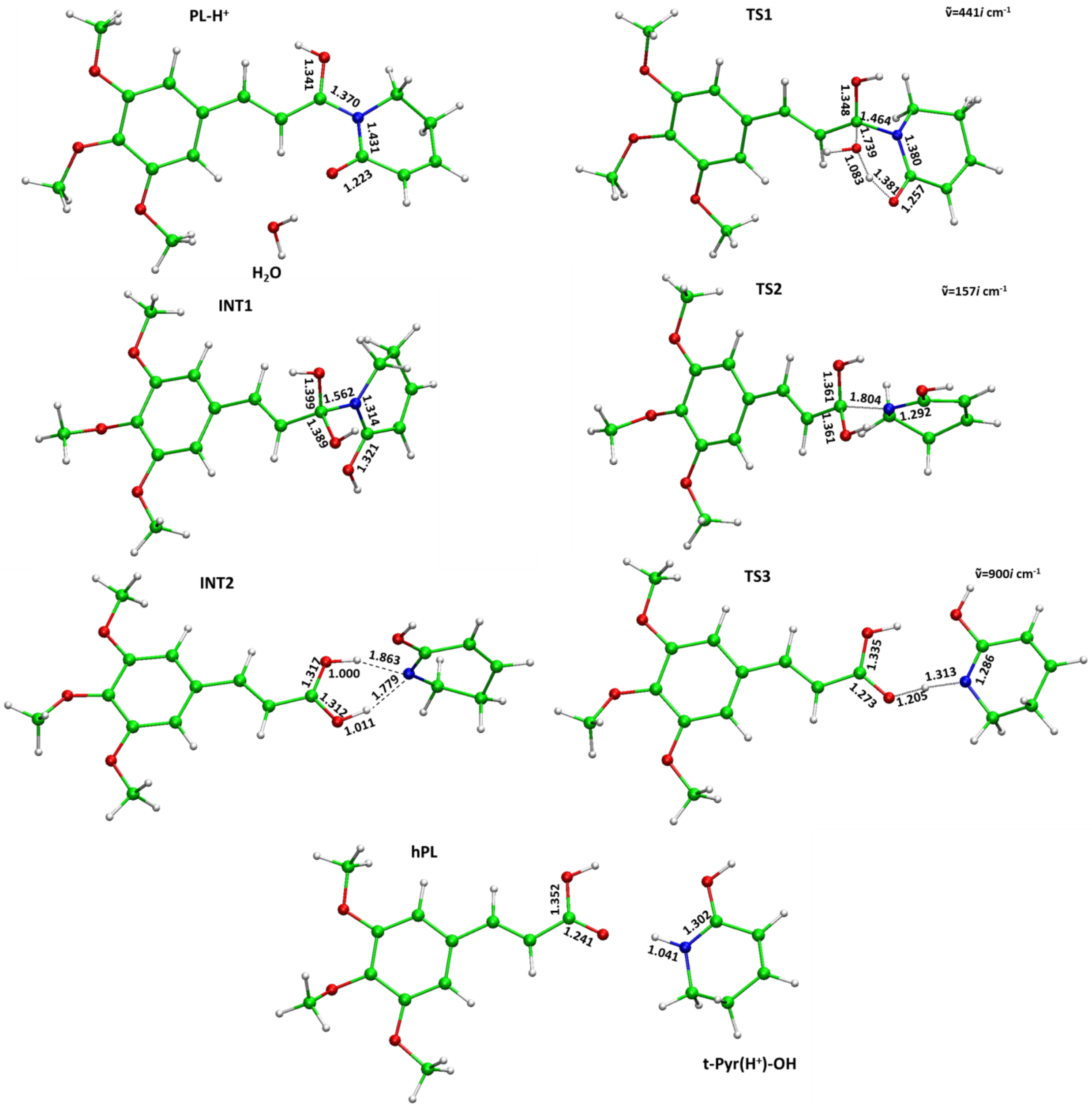

Figure S5. Optimized geometries at B3LYP/6-31+G(d,p) level of theory for hydrolysis of PL in acidic conditions.

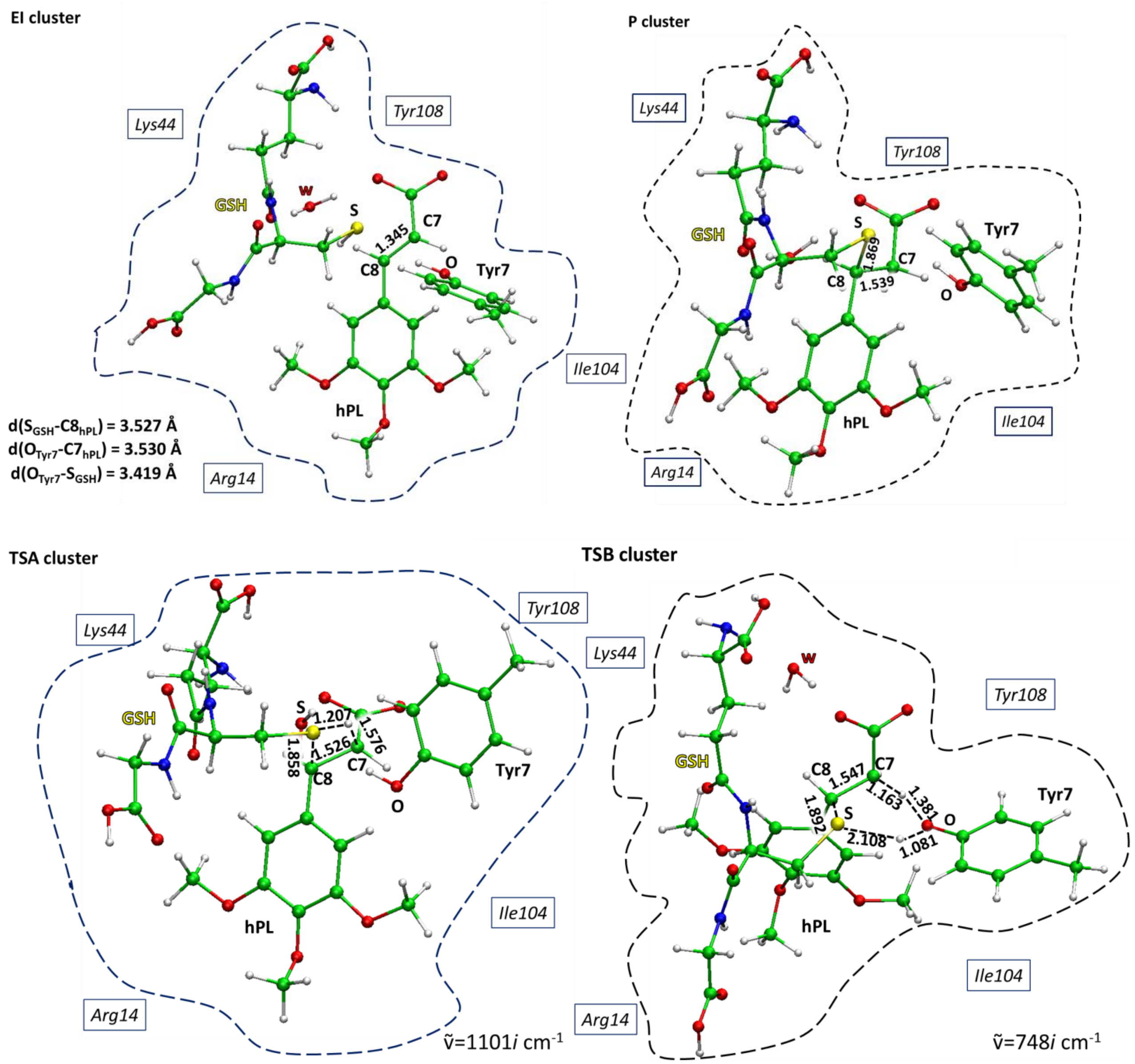

Figure S6. QM optimized geometries at B3LYP/6-31+G(d,p) level of theory of stationary points intercepted for A and B mechanisms. For clarity, only the residues directly involved in the reaction are represented in ball and stick. Imaginary frequencies of transition states are also reported.

Table S1. Calculated parameters for hPL inhibitor.

Atomtype	Charge
C	0.616
O2	0.434
CD	0.360
HA	0.135
CA	0.360
OS	0.465
CT	0.878
H1	0.135

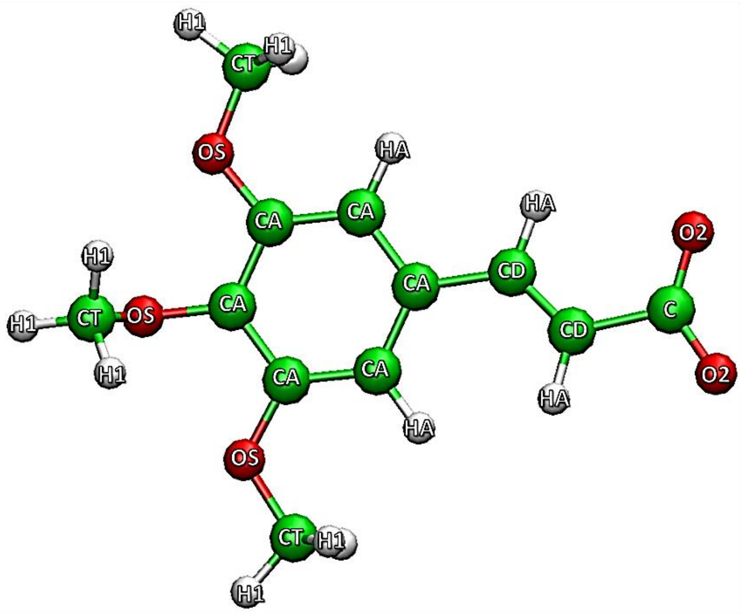

Bond	K_i / kcal mol^{-1} Å^{-2}	l_0/Å
C -O2	648.00	1.214
C -CD	449.90	1.406
CD-HA	344.30	1.087
CD-CD	390.50	1.451
CD-CA	366.00	1.472
CA-CA	478.40	1.387

CA-HA	344.30	1.087
CA-OS	392.60	1.357
OS-CT	301.50	1.439
CT-H1	337.30	1.092

**Angle**	**K_{an}/ Kcal mol^{-1} rad^{-2}**	**θ_0/deg**
C -CD-HA	48.000	119.700
C -CD-CD	67.930	120.700
O2-C -O2	78.170	130.380
O2-C -CD	72.770	119.120
CD-CD-HA	50.300	119.700
CD-CD-CA	63.840	119.620
CD-CA-CA	64.880	120.660
HA-CD-CA	50.300	119.700
CA-CA-HA	50.300	119.700
CA-CA-CA	67.180	119.970
CA-CA-OS	71.040	121.890
CA-OS-CT	64.210	112.090
OS-CT-H1	50.870	108.700
H1-CT-H1	39.430	108.350

Dihedral	d	V_n/ Kcal mol^{-1} rad^{-2}	γ/deg	n
C -CD-CD-HA	1	6.650	180.000	2.000
C -CD-CD-CA	1	6.650	180.000	2.000
O2-C -CD-HA	1	2.175	180.000	2.000
O2-C -CD-CD	1	2.175	180.000	-2.000
O2-C -CD-CD	1	0.300	0.000	3.000
CD-CD-CA-CA	1	6.650	180.000	2.000
HA-CD-CD-HA	1	6.650	180.000	2.000
HA-CD-CD-CA	1	6.650	180.000	2.000
CD-CA-CA-HA	1	6.650	180.000	2.000
CD-CA-CA-CA	1	3.625	180.000	2.000
HA-CD-CA-CA	1	6.650	180.000	2.000
CA-CA-CA-OS	1	6.650	180.000	2.000
CA-CA-CA-CA	1	3.625	180.000	2.000
CA-CA-CA-HA	1	6.650	180.000	2.000
CA-CA-OS-CT	1	1.050	180.000	2.000
HA-CA-CA-OS	1	6.650	180.000	2.000
CA-OS-CT-H1	1	0.383	0.000	3.000
OS-CA-CA-OS	1	6.650	180.000	2.000

Improper	V_n/ Kcal mol^{-1} rad^{-2}	γ/deg	n
CD-O2-C -OS	1.1	180.0	2.0
C -CD-CD-HA	1.1	180.0	2.0
CA-CD-CD-HA	1.1	180.0	2.0
CA-CA-CA-CD	1.1	180.0	2.0
CA-CA-CA-HA	1.1	180.0	2.0
CA-CA-CA-OS	1.1	180.0	2.0

VdW	r_0 /Å	ε/ kcal mol^{-1}
C	1.9080	0.0860
O2	1.6612	0.2100
CD	1.9080	0.0860
HA	1.4870	0.0157
CA	1.9080	0.0860
OS	1.6837	0.1700
CT	1.9080	0.1094
H1	1.4870	0.0157

Table S2. Extrapolated *–TΔS* contributes, according with Grimme's procedure [a] for the pathways **A** and **B**. All values are in kcal mol^{-1}

ONIOM	**$\Delta E_{B3LYP-D3}$**	**Δ ZPE**	**-TΔS**	**ΔE_{TOT}**	
				**No TΔS**	**-TΔS**
ES	0.0	0.0	0.0	0.0	0.0
TSA	67.1	-2.3	-0.6	64.4	63.8
TSB	21.8	-2.0	-0.1	19.8	19.7
P	-14.6	3.8	-1.1	-10.8	-11.9

Table S3. NBO charge distributions on atoms directly involved in reactions, calculated for QMMM model. Values are in ($|e^-|$).

	EI	TS_A	TS_B	P
S_{GSH}	-0.11 (-0.08)[a]	0.37	0.22	0.17
H_{GSH}	0.13 (0.12)[a]	0.18	0.47	0.49
$C7_{hPL}$	-0.29 (-0.36)[a]	-0.65	-0.64	-0.57
$C8_{hPL}$	-0.23 (-0.14)[a]	-0.33	-0.33	-0.35
O_{Tyr7}	-0.75 (-0.74)[a]	-0.73	-0.74	-0.71
H_{Tyr7}	0.51 (0.48)[a]	0.49	0.34	0.26

[a]Calculated for stand-alone species.

Table S4. Interatomic distances between the residues included in QM region and GSH (top) and hPL (bottom) during the investigated inhibition process. All distances are in (Å). Average ($\bar{d}$) and deviation standard values (σ) are also reported, according with definition $\sigma = \sqrt{\frac{\sum_{i=1}^{N}(d_i - \bar{d})^2}{N-1}}$.

	EI	TSA	TSB	P	$\bar{d}$±σ
Tyr7	3.904	5.571	2.840	2.936	3.813 ± 1.267
Arg13	2.856	3.437	2.886	2.833	3.003 ± 0.290
Lys44	2.770	2.777	2.797	2.779	2.781 ± 0.012
Ile104	7.672	7.519	7.681	7.630	7.648 ± 0.074
Tyr108	9.477	9.575	9.200	9.040	9.323 ± 0.247

	EI	TSA	TSB	P	$\bar{d}$±σ
Tyr7	2.596	2.503	3.349	3.375	2.956 ± 0.471
Arg13	3.648	5.899	2.866	2.800	3.803 ± 1.449
Lys44	9.320	8.860	9.592	9.382	9.829 ± 0.308
Ile104	4.883	5.127	4.624	5.802	5.109 ± 0.506
Tyr108	3.675	3.825	3.935	4.019	3.864 ± 0.149

QM layer from optimized structures$_{EI}$

C	42.50804900	58.46945400	32.14840300
H	43.30026526	57.89134801	32.57628950
H	42.83146386	59.48200321	32.02573710
C	41.30204900	58.43181200	33.06657000
C	40.76152600	57.21832600	33.52742400
H	41.27334500	56.29138800	33.29334300
C	39.57442200	57.16349200	34.25756700
H	39.17434400	56.20400500	34.56892800
C	38.88460500	58.34706900	34.56984800
O	37.66683400	58.37781300	35.17280700
H	37.55357800	57.66376200	35.88304600
C	39.45367800	59.56666100	34.19008000
H	38.95539000	60.47641300	34.49477700
C	40.62522100	59.60187500	33.43803500
H	41.00534700	60.56336600	33.10701300
C	38.86053500	54.00413900	39.57193100
H	38.45223568	53.94209143	38.58484349
H	39.77323206	53.44741649	39.61602337
N	37.90318600	53.44930800	40.53218400
H	36.93057300	53.59519000	40.29577300
C	38.18682200	53.31702000	41.85261300
N	39.42937600	53.13825600	42.26068800
H	40.16540300	52.70518000	41.69194600
H	39.64975300	53.20249900	43.26446900
N	37.17845500	53.36382300	42.74574300
H	36.36293900	53.92224800	42.54405200
H	37.42281800	53.17943000	43.73412800
C	33.18133600	54.89116600	41.71703200
H	33.42077215	54.10069648	42.39727333
C	32.94797300	54.30027300	40.31225200
H	32.50114100	55.02582600	39.62230400
H	33.90082300	53.99433900	39.86287200
H	32.29342800	53.41409500	40.32456700
C	34.34766800	55.91304200	41.68892900
H	34.60915200	56.18780800	42.71365200
H	35.22602400	55.40279400	41.26190100
C	34.12895000	57.19365200	40.86941500
H	33.32068600	57.81266200	41.27207300
H	35.04359900	57.80000400	40.87726500
H	33.91777900	56.96951600	39.81977400
C	28.49453100	54.38548500	37.88757300
H	29.10535808	54.08972526	38.71480722
H	28.20515082	53.51918298	37.33018631
C	29.27468100	55.32106200	36.99485000
C	28.96488900	55.41297400	35.63265300
H	28.18460800	54.77730500	35.21310200
C	29.65063100	56.29793700	34.79377400
H	29.44421900	56.33084900	33.72392300
C	30.66430300	57.10823000	35.31664100
O	31.35390600	57.99979900	34.53667000
H	31.25201700	57.75232700	33.59753700
C	30.98610800	57.02739900	36.67473300
H	31.79093200	57.63983000	37.06800900
C	30.29856500	56.13716400	37.49543000
H	30.56572900	56.06619000	38.54615300
C	36.17841700	56.23376900	37.47548900
O	35.84043400	55.70363100	38.54142600
O	37.29846100	56.81471700	37.21332500
C	35.25992100	56.14046700	36.27258200
H	35.46768000	56.83089000	35.45518900
C	34.35812900	55.15533800	36.13841900
H	34.24700300	54.46300700	36.97137200
C	33.58761300	54.87224500	34.92190500
C	33.05091900	53.58483900	34.72368100
H	33.18360000	52.83930100	35.50095100
C	32.37729500	53.26421600	33.54626400
O	31.90959900	52.00509300	33.24623300
C	31.75734400	51.07711700	34.32084000
H	31.16991100	51.51714900	35.13563200
H	31.21844300	50.22761800	33.89503000
H	32.71751500	50.72432600	34.71875500
C	32.17038500	54.24538000	32.55320300
O	31.44843500	53.87325400	31.43946900
C	31.91454000	54.30684500	30.16129100
H	31.59205100	55.32872600	29.95710200
H	33.00738100	54.24643700	30.09833000
H	31.47330700	53.62841200	29.42465800
C	32.63153300	55.54816000	32.79324600
O	32.20325300	56.55517500	31.93948900
C	33.21564000	57.37899800	31.34311000
H	33.87107900	57.82947400	32.09530200
H	32.68088100	58.16130000	30.80109900
H	33.83052200	56.79840100	30.64875300
C	33.37172300	55.84104300	33.93500900
H	33.69297900	56.85990400	34.10755200
N	40.51964500	48.67879400	40.63217500
H	41.32739500	48.05490300	40.47744200
H	39.66788400	48.11914200	40.84275800
H	40.73688800	49.30451900	41.44048500
C	40.37340600	49.63548600	39.47777500
C	40.94824900	50.99719600	39.98644700
O	41.15685800	51.88153200	39.12879000
O	41.12324500	51.08043200	41.23488500
H	41.00217400	49.26069200	38.66853400
C	38.91449400	49.75071800	38.97162200
H	38.32565900	50.37926000	39.64840200
H	38.45316900	48.75588500	38.96300400
C	38.93068600	50.35240000	37.55909100
H	39.58375000	51.23578200	37.57011700
H	39.40008600	49.64695200	36.86250000
C	37.60348000	50.79695400	36.96799400
O	36.57429700	50.99531600	37.62251100
N	37.65782400	50.99749700	35.60283300
H	38.59860500	50.96664000	35.20732700
C	36.68472200	51.77663500	34.87742000
C	37.09323500	53.28414500	34.66329800
S	38.41385900	53.85749200	35.80490100
H	37.84343200	55.02583400	36.17700000
H	36.21825100	53.91996100	34.79594900
H	37.45101300	53.41380800	33.64026600
H	35.76497300	51.72594500	35.46496700
C	36.45103500	51.24662500	33.44669800
O	37.36393900	50.89858200	32.69838100
N	35.16527800	51.32573700	32.99277100
H	34.47068400	51.71524800	33.61302000
C	34.85399900	51.30454100	31.57645300
H	33.94584300	51.89199200	31.41513600
H	35.67469500	51.79599600	31.04155200
C	34.65085400	49.94172500	30.88365500
O	34.80555000	48.86919300	31.51893800
O	34.38354500	50.01288400	29.64034100
O	34.59337000	49.28452300	36.81350200
H	35.38304500	48.74444600	36.55724000
H	35.05835100	50.03365200	37.24335800
C	35.96259700	46.85959500	28.17090200
H	34.94058665	46.75426464	27.87207035
H	36.33641023	45.91360766	28.50297371
N	36.05721800	47.86238300	29.29774100
H	35.76534600	48.83260800	29.03907800
H	35.44777500	47.68609900	30.12729100
H	37.02500800	47.90300700	29.68118200
H	36.54380652	47.20098186	27.33990831
H	42.24350221	58.06157505	31.19522423
H	27.62035865	54.88470344	38.25020733
H	39.05532637	55.02812367	39.81361614
H	32.28947602	55.38225999	42.04614059

TSA

C	42.60120800	57.99151600	32.35994000
H	43.20377800	57.20050165	32.75504213
H	43.18091959	58.88933126	32.30738297
C	41.40441100	58.21485500	33.26361500
C	40.69061700	57.19518300	33.91677100
H	41.02486400	56.16483700	33.82618000
C	39.54758500	57.45842000	34.68388700
H	39.00677100	56.65735600	35.18120200

C	39.06371700	58.77643600	34.80635500
O	37.93393700	59.13633300	35.43093900
H	37.47694000	58.37758300	35.95620300
C	39.80167800	59.80249800	34.20027500
H	39.44513300	60.81118400	34.33097100
C	40.93267400	59.52382300	33.45233500
H	41.45720300	60.33649400	32.95983100
C	38.71678500	53.93856000	39.21081900
H	38.26325444	53.79568330	38.25228038
H	39.68342001	53.47983031	39.21997523
N	37.87736900	53.32864800	40.24365200
H	37.13415500	52.72048200	39.92541900
C	38.25727500	53.18977700	41.54330400
N	39.50140800	53.42377100	41.92321000
H	40.28886000	53.28455700	41.28364900
H	39.74522800	53.44315000	42.92705900
N	37.31076400	52.84271600	42.43446700
H	36.36350300	53.12973800	42.23732400
H	37.57873200	52.79507400	43.43398300
C	33.19339000	54.73553100	41.75099200
H	33.36003318	53.95225897	42.46065036
C	32.97318300	54.12119700	40.35498400
H	32.66032000	54.86915200	39.61759200
H	33.91025500	53.68221700	39.98283400
H	32.22473500	53.31313700	40.34412800
C	34.42321000	55.67959200	41.72978200
H	34.65468600	55.98867400	42.74968300
H	35.28768500	55.08109300	41.39262400
C	34.33857000	56.93394900	40.84589400
H	33.52718400	57.60104400	41.15724600
H	35.27177800	57.50590100	40.93172500
H	34.21401200	56.71972300	39.77961000
C	28.53358300	54.36452900	37.86035200
H	29.13660394	54.06396492	38.69157217
H	28.22467775	53.49898151	37.31235100
C	29.33828500	55.27415500	36.95567300
C	28.95540400	55.45043200	35.61992300
H	28.09168200	54.91008400	35.22999200
C	29.68037200	56.29114200	34.76686100
H	29.41131800	56.38558600	33.71368400
C	30.80916200	56.96711700	35.24411000
O	31.53519400	57.81670000	34.45039400
H	31.10054200	57.90551400	33.58118100
C	31.22466900	56.78638100	36.56843200
H	32.14374200	57.25928900	36.91112100
C	30.48520100	55.94794100	37.40293900
H	30.81156000	55.80699500	38.43008000
C	35.68065300	56.93503600	36.81902700
O	34.61449800	57.31183200	37.36506300
O	36.84168600	57.48474500	36.96250500
C	35.70687100	55.66916300	36.04623400
H	36.35635000	55.65219900	35.16993700
C	34.39423800	54.86270500	35.94805900
H	33.70210600	55.29637900	36.67988200
C	33.69863500	54.53182200	34.65197400
C	33.00959000	53.31831000	34.49066200
H	32.95865600	52.62516800	35.32202500
C	32.42493100	52.99992000	33.26433600
O	31.85332100	51.77500200	32.98764800
C	31.47222100	50.95909500	34.08964500
H	30.86117600	51.52203000	34.80557600
H	30.87937400	50.14697300	33.66183100
H	32.34036600	50.53568300	34.61193100
C	32.43333400	53.91966200	32.20649700
O	31.72186700	53.63142800	31.05402000
C	32.49848900	53.40648700	29.86989600
H	33.27275100	54.16957500	29.75835400
H	32.95795100	52.40864600	29.87769100
H	31.80337100	53.48028200	29.02788700
C	33.07675900	55.15907300	32.38810700
O	32.98282600	56.01841400	31.33383200
C	33.58169200	57.30405000	31.47436900
H	33.25504300	57.77843500	32.40368300
H	33.23990200	57.88682900	30.61745500
H	34.67222200	57.23528700	31.46322800
C	33.74948900	55.43580600	33.58586500
H	34.30022000	56.35811600	33.72003900
N	40.82000000	48.52006900	40.74879100
H	41.49610100	47.81000500	40.43047700
H	39.92646800	48.06403700	41.03082700
H	41.23912800	49.02367800	41.56516300
C	40.63778300	49.60802800	39.71930700
C	41.17395800	50.92625000	40.38746300
O	41.09257100	51.97727200	39.69242100
O	41.67715800	50.79320500	41.52528800
H	41.30573700	49.36244600	38.88942700
C	39.19078100	49.67587300	39.18858300
H	38.54727200	50.18081300	39.91542800
H	38.79716900	48.65956900	39.07142600
C	39.15020800	50.41329200	37.83978700
H	39.69155500	51.36181600	37.95045100
H	39.69178400	49.83992800	37.07772400
C	37.75912900	50.73088100	37.31935500
O	36.77653900	50.90525400	38.06342700
N	37.66122800	50.84700000	35.96006000
H	38.54055000	51.00299800	35.46408800
C	36.47136700	51.35353500	35.30248300
C	36.30313100	52.87968100	35.46480600
S	35.15985500	53.38613500	36.82418800
H	35.90873000	54.48794200	36.89337200
H	35.88932000	53.32235300	34.55812800
H	37.27076700	53.35463000	35.65054300
H	35.61002700	50.82834600	35.72200000
C	36.50348300	51.09024400	33.78446200
O	37.52976200	50.94768900	33.12740700
N	35.27711100	51.12001800	33.18936500
H	34.47187400	51.33086000	33.76198200
C	35.11269800	51.20677200	31.75445000
H	34.39385200	51.99472000	31.52016600
H	36.08224900	51.50780500	31.34179300
C	34.70039400	49.91226600	31.03143100
O	34.84737400	48.81515100	31.62676200
O	34.32806800	50.03293600	29.81998800
O	34.81554400	49.19647600	37.04865300
H	35.61231600	48.69112000	36.74675000
H	35.24563200	49.88715700	37.59456600
C	35.87243300	46.83976700	28.16742700
H	34.85130984	46.72652390	27.86845628
H	36.25137578	45.89823671	28.50628254
N	35.96042300	47.85223400	29.28908300
H	35.66454700	48.82120900	29.03306800
H	35.37232600	47.66562300	30.13212200
H	36.93312500	47.90650600	29.66039800
H	42.26352561	57.72710111	31.37965669
H	27.67090493	54.88794412	38.21633397
H	36.45241059	47.17879685	27.33460964
H	38.81717958	54.98592918	39.40534082
H	32.32442278	55.29176621	42.03453806

TSB

C	42.13079400	58.39997800	32.31914300
H	43.01427513	57.95681257	32.72897893
H	42.25678122	59.46109137	32.26377472
C	40.93960800	58.07602300	33.21121900
C	40.69171700	56.74057900	33.56992600
H	41.36769300	55.98203700	33.20538200
C	39.61132800	56.37009600	34.36108600
H	39.44144400	55.32487700	34.60709600
C	38.72863600	57.34753500	34.84147600
O	37.61414100	56.98871500	35.59948800
H	36.48061300	56.92096000	36.25309300
C	38.95748700	58.68749500	34.52064500
H	38.28879800	59.45367800	34.90110500
C	40.04672100	59.03836500	33.70812300
H	40.18621800	60.08808300	33.45653400
C	39.29578900	54.17528100	39.50178100
H	39.03050844	54.28471731	38.47098038
H	40.15183329	53.53864195	39.58411505
N	38.17119600	53.58100100	40.23633400
H	37.30167300	54.07170300	39.99525300
C	38.34387100	53.22146400	41.53418600
N	39.46627200	52.62200000	41.91329000
H	40.15406500	52.28103400	41.22891500
H	39.73468200	52.63148400	42.90536500

N	37.36422500	53.43209000	42.43116400
H	36.67916200	54.14654300	42.22929900
H	37.59298800	53.24927600	43.42400800
C	33.10398000	54.80740600	41.76139300
H	33.37098067	54.06937261	42.48866005
C	32.87083800	54.12052900	40.40034500
H	32.40681300	54.78802900	39.66376600
H	33.82931800	53.79763900	39.97467400
H	32.23071500	53.22628100	40.47520000
C	34.24385500	55.85288600	41.65017100
H	34.53900400	56.17034700	42.65402200
H	35.11251300	55.35191600	41.19917200
C	33.95538500	57.09883600	40.79800500
H	33.22327200	57.76366600	41.26844200
H	34.87742400	57.66245700	40.62538900
H	33.57508800	56.82713900	39.80494100
C	28.51259600	54.37115300	37.91598000
H	29.07745825	54.01830450	38.75343363
H	28.21432119	53.54025910	37.31137479
C	29.36377100	55.31210200	37.09458700
C	29.16964200	55.41483000	35.71146900
H	28.42083400	54.78682500	35.22721500
C	29.93212300	56.29455400	34.93405600
H	29.80937400	56.33145700	33.85172600
C	30.91265500	57.08625300	35.54318000
O	31.68368400	57.96565600	34.82806000
H	31.75807300	57.65880900	33.90351900
C	31.11419600	57.00024500	36.92470900
H	31.88902700	57.60424100	37.38628900
C	30.34920000	56.11779900	37.68306600
H	30.52759000	56.04126700	38.75183700
C	36.28941600	56.42948700	38.37133400
O	36.27210500	55.28990600	38.93708600
O	36.78802200	57.48835700	38.80636900
C	35.63606600	56.50717300	36.95568300
H	34.79373300	57.21213700	37.00315900
C	35.14849500	55.10913700	36.55082900
H	34.71011600	54.63010600	37.42813200
C	34.20902700	54.94276300	35.38226900
C	33.46310800	53.75085200	35.28565300
H	33.56234000	53.01372700	36.07584300
C	32.66853200	53.47720000	34.17682600
O	32.02897400	52.27541200	33.96336100
C	31.74468800	51.46080600	35.10597900
H	31.31862500	52.06409400	35.91572300
H	31.00456100	50.73260700	34.76137500
H	32.62711300	50.91978700	35.47113100
C	32.52076500	54.43858200	33.15334100
O	31.67698100	54.12343600	32.10453800
C	32.07584800	54.51811000	30.79095400
H	33.15346700	54.37719000	30.64188600
H	31.53388000	53.86757000	30.09487400

H	31.81670100	55.55970400	30.59060900
C	33.17909700	55.66516500	33.30588300
O	32.81687400	56.69176000	32.43674400
C	33.87554700	57.33095300	31.71562600
H	34.58504800	57.82656600	32.38757300
H	33.39765100	58.07451900	31.07316000
H	34.41610800	56.60531600	31.09884600
C	34.05189500	55.89711100	34.37687800
H	34.56666500	56.84830600	34.45634700
N	40.30757200	49.02595700	40.60402100
H	41.17665800	48.46467800	40.68220100
H	39.50907700	48.44334500	40.93852800
H	40.42612100	49.87310800	41.17499100
C	40.16599100	49.46951400	39.17141200
C	41.13839100	50.66989500	38.95811900
O	41.69232900	50.74757600	37.85155500
O	41.24165100	51.45950800	39.95792700
H	40.48904000	48.62748800	38.55801900
C	38.71314600	49.84780800	38.83900000
H	38.43602200	50.76713500	39.36795300
H	38.03633200	49.05121400	39.16672200
C	38.59795400	50.05226000	37.32340200
H	39.44251100	50.65199000	36.96530500
H	38.68678100	49.07739200	36.82311100
C	37.29243900	50.65645700	36.83760700
O	36.25972000	50.70312400	37.50922900
N	37.36504700	51.08573400	35.53271300
H	38.30688200	51.30177000	35.20883600
C	36.31320100	51.76533300	34.81826400
C	36.60792100	53.28288200	34.70370000
S	36.75647800	54.09458800	36.33419500
H	37.66768500	56.00468000	35.76739800
H	35.84744100	53.77517200	34.09475100
H	37.56635300	53.39475000	34.19133000
H	35.38575000	51.58822800	35.36594100
C	36.18939400	51.29400900	33.35901400
O	37.16750800	51.08523700	32.63998200
N	34.91560000	51.28644300	32.86569100
H	34.14246200	51.46436900	33.49159200
C	34.62951300	51.29934600	31.45104000
H	33.72997700	51.90020200	31.28054900
H	35.46123700	51.80149500	30.94551200
C	34.43878300	49.94939600	30.73750400
O	34.63740600	48.87168900	31.35334300
O	34.14020100	50.03792800	29.50428400
O	34.22473000	49.19913300	36.68034400
H	35.02208700	48.69886000	36.36752500
H	34.69956800	49.91162100	37.15948500
C	35.76796900	46.75785600	28.09534000
H	34.74608635	46.62687271	27.80637766
H	36.14745282	45.83881494	28.49063174
N	35.85907600	47.83839200	29.15320800

H	35.54842600	48.78705600	28.84600800
H	35.28331500	47.69274000	30.01466600
H	36.83492000	47.92005600	29.51096200
H	36.34601768	47.04468266	27.24182364
H	39.52426152	55.13540099	39.91514514
H	32.20378744	55.29729485	42.06890280
H	41.95818063	58.00962118	31.33795689
H	27.64344846	54.88878289	38.26460854
P			
C	42.41772100	58.43323100	32.18357500
H	43.27282290	57.92002706	32.57128939
H	42.62736667	59.48076951	32.12347702
C	41.22930500	58.20612300	33.10497300
C	40.89325000	56.90370200	33.50740800
H	41.52357500	56.09119400	33.17978300
C	39.78152800	56.62460700	34.29485700
H	39.56101200	55.60086400	34.58516700
C	38.93769100	57.66798400	34.71521400
O	37.79921000	57.46143300	35.42272600
H	35.35767000	57.01464100	36.61464300
C	39.28113200	58.98317300	34.36758000
H	38.65969800	59.79976300	34.72130600
C	40.40078000	59.23883100	33.57025900
H	40.61111100	60.26869200	33.28921900
C	39.14417300	54.13438400	39.50888400
H	38.80787794	54.26460755	38.50148762
H	39.98155497	53.46841320	39.52208717
N	38.05653400	53.56966800	40.31910700
H	37.16285300	54.02878600	40.08466300
C	38.26261100	53.21133000	41.60279100
N	39.41831200	52.64559900	41.97623400
H	40.16234600	52.48593100	41.27937300
H	39.72279400	52.76136000	42.95316300
N	37.27511200	53.34862500	42.50543200
H	36.54211400	54.01487400	42.30699500
H	37.52691700	53.19152100	43.49687200
C	33.08151200	54.89531300	41.80108600
H	33.34995536	54.14018447	42.51004232
C	32.85670100	54.24016600	40.42266100
H	32.34360900	54.90731800	39.71816600
H	33.81842800	53.98188800	39.96498900
H	32.25521900	53.31831400	40.48542000
C	34.21276500	55.95347200	41.73012500
H	34.47692900	56.26760900	42.74266800
H	35.09610700	55.46538900	41.29850000
C	33.92702500	57.20377300	40.88349200
H	33.18117900	57.85868800	41.34550500
H	34.84549000	57.78261600	40.73513400
H	33.56456000	56.93892700	39.88278200
C	28.51934600	54.39706000	37.93306000
H	29.08886045	54.05874029	38.77335344
H	28.22505733	53.55652744	37.33993337

C	29.36184500	55.33310300	37.09740400
C	29.17878000	55.40272100	35.71085700
H	28.43614400	54.76106300	35.23529400
C	29.94730800	56.26323700	34.91748400
H	29.83400500	56.27052700	33.83355300
C	30.92287400	57.06943500	35.51435900
O	31.71266900	57.91941800	34.78450800
H	31.76738400	57.60465600	33.86169400
C	31.10364300	57.02708100	36.90113100
H	31.86769500	57.65053200	37.35443100
C	30.33514800	56.16140000	37.67490000
H	30.50232300	56.11389900	38.74708200
C	36.07794600	56.09719100	38.46479800
O	35.97998800	55.03210400	39.14381000
O	36.82165100	57.08234000	38.63468600
C	35.10153200	56.15597900	37.24391900
H	34.08137900	56.28965800	37.62268800
C	35.14288700	54.84310900	36.45639400
H	34.98186100	54.02936900	37.16717500
C	34.17845500	54.72235100	35.29762600
C	33.41818600	53.54948400	35.15560900
H	33.50396700	52.77773300	35.91314700
C	32.59546700	53.35004400	34.04504400
O	31.92904900	52.17505300	33.78496200
C	31.73767100	51.26484700	34.87075000
H	31.29839100	51.77698100	35.73518400
H	31.03897700	50.51312600	34.49389600
H	32.66503500	50.76519800	35.18033200
C	32.46297800	54.35770400	33.06793200
O	31.60253000	54.11311000	32.01464500
C	32.03193300	54.49454900	30.70496900
H	33.09424600	54.26756300	30.55213700
H	31.43603100	53.90251200	30.00138900
H	31.86039900	55.55695000	30.52309400
C	33.16167100	55.55967700	33.25950600
O	32.85709800	56.61092400	32.40356400
C	33.96587800	57.27943400	31.78585800
H	34.58957300	57.80165100	32.51952000
H	33.53051400	58.00457000	31.09430100
H	34.58606700	56.56722300	31.23188400
C	34.03562900	55.72620800	34.33669300
H	34.58512100	56.65458700	34.44636500
N	39.87952400	49.57577500	40.90008200
H	40.84383400	49.35842500	41.21555700
H	39.25459700	48.78286000	41.16782800
H	39.59413500	50.43407400	41.38766900
C	39.90057000	49.78367600	39.40576900
C	40.96000300	50.86806100	39.04130600
O	41.47963300	50.74432000	37.91787300
O	41.15045900	51.77981900	39.90771100
H	40.20972800	48.82607700	38.98193400
C	38.49140200	50.16227300	38.91154900
H	38.19687300	51.12245900	39.35221500
H	37.77270900	49.40437300	39.24491900
C	38.47823700	50.26776900	37.38005100
H	39.39380600	50.76383200	37.03871200
H	38.50759900	49.25817900	36.94710900
C	37.26532000	50.95452100	36.78066300
O	36.16689700	51.06629600	37.33660500
N	37.47993100	51.39576300	35.49782200
H	38.45115700	51.57935000	35.24852000
C	36.48505000	52.06842400	34.70729400
C	36.81866800	53.56179800	34.44893600
S	36.93481100	54.64090000	35.90786400
H	37.59323100	56.48330300	35.53834500
H	36.08628800	53.97751600	33.75020200
H	37.80361600	53.61264800	33.97800400
H	35.54337700	51.97301900	35.24807400
C	36.35440800	51.48221200	33.28558000
O	37.32986100	51.24072300	32.57558400
N	35.07684700	51.39949000	32.80944100
H	34.31676100	51.63349200	33.43131600
C	34.77914000	51.32965500	31.39658900
H	33.88183600	51.92552200	31.19785100
H	35.61052300	51.79996500	30.86099100
C	34.57012900	49.94675100	30.75127800
O	34.74034100	48.89353200	31.41404200
O	34.28213500	49.98641200	29.51167300
O	34.29221300	49.21762500	36.75871700
H	35.11259500	48.74487300	36.46347400
H	34.71896700	49.98258600	37.19623900
C	35.89567600	46.79588700	28.08898700
H	34.88040563	46.67829347	27.77228867
H	36.27049702	45.85748785	28.44086231
N	35.96095300	47.81473500	29.20410000
H	35.64033100	48.77368500	28.93659000
H	35.36961700	47.62495000	30.04278800
H	36.92972900	47.89549300	29.58152200
H	42.18910622	58.05868261	31.20769141
H	39.43524119	55.08142826	39.91297122
H	36.48992706	47.13081359	27.26461460
H	32.17579508	55.36912355	42.11744091
H	27.64766697	54.91323387	38.27750188

QM cluster from optimized geometries

EI

C	5.47400000	2.51100000	100.35700000
C	4.55200000	2.59300000	99.15900000
C	4.14800000	3.82400000	98.62900000
C	4.09300000	1.43200000	98.51900000
C	3.32600000	3.90400000	97.50300000
C	3.27600000	1.49200000	97.39400000
C	2.88800000	2.73400000	96.87400000
O	2.08300000	2.72800000	95.77200000
C	4.25900000	6.34100000	91.57900000
N	3.48900000	5.54300000	90.62900000
C	4.02700000	4.90700000	89.58700000
N	5.15100000	5.41400000	89.00800000
N	3.46900000	3.77600000	89.16600000
C	-4.04800000	13.55400000	101.18600000
N	-3.44400000	13.28500000	99.86900000
C	-0.53300000	4.81900000	86.99500000
C	0.26500000	3.89300000	87.92600000
C	-1.67600000	5.57400000	87.68900000
C	-0.58900000	2.85300000	88.66100000
C	-6.30200000	3.93000000	89.91700000
C	-5.45100000	3.06100000	90.81500000
C	-5.94500000	2.55800000	92.02300000
C	-4.13600000	2.72400000	90.45800000
C	-5.16200000	1.75500000	92.85500000
C	-3.34200000	1.92300000	91.27200000
C	-3.85300000	1.44000000	92.48200000
O	-3.02900000	0.67300000	93.25900000
C	1.10200000	3.50000000	91.57600000
O	1.88800000	2.68200000	91.02400000
C	0.30700000	2.99700000	92.73300000
O	-5.04800000	3.85200000	95.56300000
C	-0.75700000	3.65900000	93.22100000
O	-4.25400000	1.96700000	97.29400000
C	-1.59600000	3.26700000	94.35300000
O	-1.71600000	1.09700000	97.34800000
C	-2.88100000	3.82300000	94.42800000
O	0.98000000	4.73100000	91.22200000
C	-3.76900000	3.40100000	95.42200000
C	-5.47000000	4.91100000	94.71100000
C	-3.36500000	2.43000000	96.35600000
C	-4.22900000	2.69800000	98.52100000
C	-2.03900000	1.94800000	96.33300000
C	-0.37600000	0.61800000	97.40400000
C	-1.16100000	2.35400000	95.32700000
N	1.36700000	7.34800000	88.33300000
C	1.63100000	8.42900000	89.30900000
C	3.15800000	8.64100000	89.36000000
O	3.81300000	7.99800000	88.37300000
O	3.73300000	9.31000000	90.19100000
C	1.01500000	8.12300000	90.68000000
C	1.05200000	9.24700000	91.72300000
C	0.46200000	8.72000000	93.02400000
O	-0.59800000	8.08700000	93.06400000
N	1.18700000	8.97100000	94.14500000
C	0.81300000	8.44900000	95.44700000
C	1.14900000	9.54000000	96.47700000
O	2.27900000	10.02300000	96.50500000
C	1.59400000	7.17100000	95.83100000
S	1.65400000	5.88100000	94.52000000
N	0.15800000	9.90300000	97.32300000
C	0.33700000	10.98800000	98.26700000
C	-1.01800000	11.53700000	98.68800000
O	-2.07500000	11.04600000	98.31200000
O	-0.89800000	12.57700000	99.49200000
H	5.19300000	1.68700000	101.02400000
H	6.51500000	2.33900000	100.05200000
H	4.48100000	4.74500000	99.10400000
H	4.38400000	0.45900000	98.90800000
H	3.02400000	4.87300000	97.11300000
H	2.93200000	0.58900000	96.89800000
H	1.89500000	3.64200000	95.47800000
H	3.60000000	6.56000000	92.42100000
H	4.59900000	7.28900000	91.15500000
H	2.53600000	5.21400000	90.91300000
H	5.19000000	6.42700000	88.93200000
H	5.54400000	4.90900000	88.22700000
H	2.77900000	3.28300000	89.81200000
H	3.67100000	3.41200000	88.24700000
H	-3.65700000	14.49900000	101.57300000
H	-5.14500000	13.61100000	101.16500000
H	-1.84200000	12.92600000	99.72200000
H	-3.70200000	14.01100000	99.20100000
H	-3.77700000	12.40400000	99.47500000
H	0.16100000	5.53500000	86.53000000
H	-1.35400000	6.06900000	88.61200000
H	-2.48200000	4.89300000	87.98100000
H	-2.11300000	6.32700000	87.02100000
H	0.78700000	4.48000000	88.69100000
H	1.04400000	3.38500000	87.33800000
H	0.04600000	2.17900000	89.24500000
H	-1.27100000	3.34200000	89.36400000
H	-1.18800000	2.25500000	87.96200000
H	-7.18100000	4.31400000	90.44700000
H	-5.73500000	4.79000000	89.54100000
H	-6.96100000	2.80000000	92.33000000
H	-3.71700000	3.11300000	89.53200000
H	-5.55500000	1.40100000	93.80500000
H	-2.31400000	1.69700000	91.01100000
H	-3.30600000	0.74900000	94.18700000
H	0.93700000	11.78600000	97.81800000
H	0.87900000	10.66000000	99.16300000
H	-0.79800000	9.59400000	97.18500000
H	0.63000000	2.04200000	93.13700000
H	-3.19300000	4.52200000	93.66100000
H	-6.49100000	5.14700000	95.02100000
H	-4.83600000	5.80000000	94.83500000
H	-5.46900000	4.60700000	93.65900000
H	-4.97300000	2.23300000	99.17300000
H	-3.24000000	2.63500000	98.99300000
H	-4.49600000	3.75000000	98.35500000
H	-0.32500000	-0.01100000	98.29500000
H	0.34500000	1.43900000	97.49000000
H	-0.12700000	0.01800000	96.51900000
H	-0.14300000	1.99000000	95.29900000
H	0.50300000	7.51100000	87.82400000
H	1.23900000	6.46400000	88.82700000
H	1.21100000	9.36300000	88.91200000
H	-0.02400000	7.83500000	90.50600000
H	1.50000000	7.23300000	91.09000000
H	0.43500000	10.09200000	91.38700000
H	2.07100000	9.61600000	91.86100000
H	2.06900000	9.46500000	94.09600000
H	-0.25600000	8.22700000	95.40600000
H	2.63700000	7.43700000	96.02500000
H	1.17300000	6.75800000	96.75400000
H	-0.94600000	4.22600000	86.16600000
H	-6.66400000	3.37500000	89.04100000
H	5.12600000	5.77900000	91.94500000
H	-3.75500000	12.76000000	101.87800000
H	5.45500000	3.43700000	100.94200000
H	-1.08000000	4.55600000	92.69800000
H	0.34900000	5.88700000	94.19300000
H	3.06900000	7.52500000	87.88800000
O	-1.48700000	6.13500000	91.07000000
H	-0.64800000	5.62600000	91.08100000
H	-1.33800000	6.81900000	91.75000000

TSA

C	5.47300000	2.49900000	100.34800000
C	4.16100000	3.11900000	99.92700000
C	4.11900000	4.20800000	99.05200000
C	2.93700000	2.62900000	100.40800000
C	2.91200000	4.79400000	98.66600000
C	1.72300000	3.19800000	100.03300000
C	1.70400000	4.28800000	99.15400000
O	0.48600000	4.81400000	98.81600000
C	4.26300000	6.33300000	91.57100000
N	3.12800000	5.42100000	91.53700000
C	2.95200000	4.47100000	90.62700000
N	3.68100000	4.51100000	89.47200000
N	2.12800000	3.45300000	90.88600000

C	-4.04100000	13.54900000	101.17900000
N	-2.77300000	13.69500000	101.92300000
C	-0.77900000	4.53200000	86.99000000
C	0.26900000	3.89000000	87.91600000
C	-2.21400000	4.06000000	87.25400000
C	0.47100000	2.38900000	87.65800000
C	-6.29900000	3.93100000	89.90500000
C	-6.43200000	3.87900000	91.41200000
C	-6.44600000	5.04400000	92.18700000
C	-6.52200000	2.65400000	92.09000000
C	-6.53400000	5.00400000	93.58100000
C	-6.60800000	2.59100000	93.47800000
C	-6.60700000	3.76900000	94.23900000
O	-6.68800000	3.64300000	95.59100000
C	0.61800000	4.69500000	93.62300000
O	0.94000000	3.50900000	93.38300000
C	0.14200000	5.05400000	95.00100000
O	-5.28800000	7.97400000	95.19600000
C	-0.64400000	6.36400000	95.16300000
O	-6.00400000	6.07100000	96.93700000
C	-2.01000000	6.36000000	95.78000000
O	-4.15600000	4.56400000	98.19600000
C	-2.96000000	7.23200000	95.23700000
O	0.73100000	5.67400000	92.78800000
C	-4.28800000	7.17800000	95.67300000
C	-4.95800000	8.90100000	94.16600000
C	-4.66400000	6.23900000	96.64400000
C	-6.46000000	6.69300000	98.14400000
C	-3.69500000	5.40500000	97.23000000
C	-3.24800000	3.61200000	98.74100000
C	-2.36600000	5.46700000	96.79900000
N	-0.20200000	7.73900000	88.88400000
C	0.54700000	8.70900000	89.71000000
C	2.04800000	8.37400000	89.56800000
O	2.28800000	7.38300000	88.69200000
O	2.93300000	8.93600000	90.18000000
C	0.07600000	8.71200000	91.17700000
C	0.65100000	9.80100000	92.09400000
C	-0.02600000	9.69100000	93.45700000
O	-1.24700000	9.54200000	93.55600000
N	0.80200000	9.72100000	94.54100000
C	0.28600000	9.76900000	95.89600000
C	0.68100000	11.12500000	96.51400000
O	1.74700000	11.66700000	96.22900000
C	0.82900000	8.66500000	96.82800000
S	0.86100000	6.87600000	96.12800000
N	-0.19800000	11.62600000	97.41100000
C	0.07800000	12.79100000	98.22000000
C	-0.95200000	12.84800000	99.33700000
O	-1.81000000	11.98400000	99.47000000
O	-0.81500000	13.90800000	100.11000000
H	5.69000000	2.68600000	101.40800000
H	5.47000000	1.41100000	100.20900000
H	5.04900000	4.61300000	98.65800000
H	2.93200000	1.78000000	101.08900000
H	2.91200000	5.63900000	97.98400000
H	0.78100000	2.80800000	100.40800000
H	0.60900000	5.53500000	98.16400000
H	4.39600000	6.66100000	92.60500000
H	4.11600000	7.21900000	90.94500000
H	2.31400000	5.57100000	92.16700000
H	3.78000000	5.43200000	89.05500000
H	3.53300000	3.76100000	88.81100000
H	1.65300000	3.40900000	91.82400000
H	1.69300000	2.96800000	90.11300000
H	-3.90000000	12.77300000	100.42400000
H	-4.89400000	13.28800000	101.81900000
H	-1.54600000	13.87800000	100.84000000
H	-2.56100000	12.82500000	102.41000000
H	-2.85300000	14.41900000	102.63500000
H	-0.73300000	5.62000000	87.12000000
H	-2.49700000	4.25400000	88.29600000
H	-2.33500000	2.98600000	87.07000000
H	-2.92700000	4.58300000	86.60600000
H	-0.03500000	4.06700000	88.95400000
H	1.22600000	4.41200000	87.77100000
H	1.26300000	1.95600000	88.28500000
H	-0.44000000	1.81600000	87.86100000
H	0.75500000	2.20200000	86.61500000
H	-6.59200000	4.91100000	89.51200000
H	-5.26600000	3.74500000	89.57800000
H	-6.39100000	6.01300000	91.69400000
H	-6.52200000	1.72700000	91.52000000
H	-6.54600000	5.92500000	94.15600000
H	-6.67400000	1.63800000	93.99400000
H	-6.56800000	4.51000000	96.03000000
H	0.02900000	13.71900000	97.63500000
H	1.08800000	12.74600000	98.64500000
H	-1.03400000	11.11500000	97.67400000
H	-0.18400000	4.21000000	95.60500000
H	-2.65200000	7.93500000	94.47200000
H	-5.88200000	9.44000000	93.95000000
H	-4.18300000	9.60600000	94.48700000
H	-4.61300000	8.38500000	93.26100000
H	-7.52700000	6.47100000	98.21800000
H	-5.93600000	6.28400000	99.01300000
H	-6.31300000	7.77800000	98.09000000
H	-3.82100000	3.04900000	99.48000000
H	-2.39300000	4.09700000	99.22500000
H	-2.88200000	2.92800000	97.96500000
H	-1.62000000	4.81700000	97.23700000
H	-0.96300000	8.18300000	88.37800000
H	-0.59500000	6.99000000	89.47300000
H	0.42200000	9.71300000	89.28300000
H	-1.01300000	8.82500000	91.17000000
H	0.28200000	7.73300000	91.62100000
H	0.42600000	10.79800000	91.68800000
H	1.73800000	9.71900000	92.17000000
H	1.75400000	10.04700000	94.42300000
H	-0.80000000	9.67800000	95.81200000
H	1.88400000	8.85600000	97.06300000
H	0.26600000	8.67200000	97.76600000
H	-0.51100000	4.33400000	85.94100000
H	-6.92800000	3.17600000	89.41800000
H	5.16800000	5.80600000	91.25600000
H	-4.26300000	14.49200000	100.67000000
H	6.30800000	2.90400000	99.76700000
H	-0.64500000	6.92500000	94.23100000
H	1.07400000	5.68900000	95.61500000
H	1.36200000	7.17000000	88.35000000
O	-0.98200000	5.51600000	90.66300000
H	-0.31700000	5.50200000	91.39900000
H	-1.83600000	5.50000000	91.12000000

TSB

C	5.47400000	2.51100000	100.35700000
C	4.31900000	3.02200000	99.52400000
C	3.93100000	4.36600000	99.57200000
C	3.61100000	2.17400000	98.65800000
C	2.88400000	4.85200000	98.78800000
C	2.56500000	2.64100000	97.86500000
C	2.19800000	3.98900000	97.92900000
O	1.15700000	4.42500000	97.13000000
C	4.25900000	6.34100000	91.57900000
N	3.19800000	5.34600000	91.57800000
C	3.09300000	4.35500000	90.70400000
N	3.81100000	4.41000000	89.54200000
N	2.34700000	3.28900000	91.00900000
C	-4.04800000	13.55400000	101.18600000
N	-2.76200000	13.66400000	101.90500000
C	-1.22000000	3.95400000	87.53300000
C	0.26500000	3.89300000	87.92600000
C	-2.12700000	3.04400000	88.36900000
C	0.91500000	2.53100000	87.64400000
C	-6.30200000	3.93000000	89.91700000
C	-6.42500000	3.94100000	91.42600000
C	-6.42400000	5.13600000	92.15500000
C	-6.52100000	2.74500000	92.15400000
C	-6.50400000	5.15200000	93.55000000
C	-6.60000000	2.73800000	93.54300000
C	-6.58500000	3.94500000	94.25700000
O	-6.66100000	3.87300000	95.61300000
C	0.70800000	4.52400000	93.69100000
O	1.17200000	3.37500000	93.50700000

C	-0.08200000	4.78400000	94.96600000
O	-5.15600000	8.08400000	95.06800000
C	-0.57900000	6.24400000	95.09100000
O	-5.93400000	6.31900000	96.92200000
C	-1.93300000	6.38900000	95.74600000
O	-4.14100000	4.84100000	98.28300000
C	-2.85500000	7.25900000	95.15100000
O	0.84800000	5.50200000	92.85700000
C	-4.18200000	7.29000000	95.59700000
C	-4.82000000	8.88000000	93.93300000
C	-4.58800000	6.43400000	96.63000000
C	-6.38600000	7.04100000	98.07200000
C	-3.64700000	5.60800000	97.27000000
C	-3.28700000	3.86200000	98.85800000
C	-2.31700000	5.59300000	96.83500000
N	-0.19400000	7.38400000	88.79900000
C	0.48600000	8.45500000	89.55600000
C	2.00600000	8.19700000	89.45900000
O	2.31400000	7.16200000	88.66000000
O	2.84600000	8.85300000	90.04000000
C	-0.00900000	8.54000000	91.01200000
C	0.48300000	9.72800000	91.85100000
C	-0.29300000	9.73400000	93.16300000
O	-1.52400000	9.65300000	93.17700000
N	0.45300000	9.78400000	94.30300000
C	-0.15800000	9.90900000	95.60500000
C	0.34400000	11.21200000	96.25200000
O	1.44300000	11.68500000	95.97700000
C	0.13300000	8.73700000	96.61100000
S	0.82500000	7.18900000	95.93800000
N	-0.49400000	11.74500000	97.17100000
C	-0.12300000	12.84600000	98.03100000
C	-1.08000000	12.86700000	99.21200000
O	-1.95300000	12.01800000	99.35000000
O	-0.86800000	13.87900000	100.03000000
H	5.26600000	1.51600000	100.76700000
H	6.39500000	2.42900000	99.76400000
H	4.45400000	5.05100000	100.23600000
H	3.88700000	1.12300000	98.59800000
H	2.59700000	5.90000000	98.84600000
H	2.03200000	1.97900000	97.18900000
H	1.10200000	5.50400000	97.17400000
H	4.35400000	6.72300000	92.59800000
H	4.05800000	7.18600000	90.91300000
H	2.37600000	5.44600000	92.22000000
H	3.83200000	5.32100000	89.09300000
H	3.72000000	3.62700000	88.90900000
H	1.88500000	3.25200000	91.95600000
H	1.96400000	2.72500000	90.26200000
H	-3.94500000	12.77600000	100.42900000
H	-4.89500000	13.31900000	101.84400000
H	-1.56500000	13.83600000	100.79400000
H	-2.56300000	12.78900000	102.38800000
H	-2.80800000	14.39000000	102.61900000
H	-1.56200000	4.99100000	87.64200000
H	-2.02700000	3.28400000	89.43300000
H	-1.88200000	1.98400000	88.23400000
H	-3.17800000	3.17100000	88.08600000
H	0.35300000	4.14600000	88.98800000
H	0.81000000	4.66600000	87.36700000
H	1.99500000	2.54300000	87.85300000
H	0.47200000	1.73000000	88.24600000
H	0.80400000	2.24700000	86.59000000
H	-6.52400000	4.91500000	89.49200000
H	-5.28900000	3.65400000	89.59200000
H	-6.36400000	6.08400000	91.62300000
H	-6.53200000	1.79600000	91.62100000
H	-6.50200000	6.09500000	94.09000000
H	-6.67100000	1.80700000	94.09700000
H	-6.53900000	4.75600000	96.01900000
H	-0.17200000	13.81100000	97.51000000
H	0.90900000	12.73600000	98.38400000
H	-1.34800000	11.26800000	97.44100000
H	-0.95300000	4.11800000	94.94100000
H	-2.53900000	7.88200000	94.32200000
H	-5.72800000	9.43300000	93.68600000
H	-4.00400000	9.57800000	94.14900000
H	-4.52800000	8.25200000	93.08200000
H	-7.45900000	6.85200000	98.15000000
H	-5.88200000	6.68500000	98.97700000
H	-6.21200000	8.11500000	97.94100000
H	-3.89700000	3.33000000	99.59000000
H	-2.42900000	4.32200000	99.36600000
H	-2.92300000	3.15600000	98.10200000
H	-1.59300000	4.93000000	97.29400000
H	-0.96300000	7.74700000	88.24500000
H	-0.56600000	6.67200000	89.44600000
H	0.31200000	9.41600000	89.05400000
H	-1.10200000	8.59300000	90.97800000
H	0.24300000	7.61000000	91.53100000
H	0.26600000	10.67300000	91.33400000
H	1.56300000	9.68100000	92.00800000
H	1.43400000	10.02600000	94.24600000
H	-1.23300000	9.95200000	95.42500000
H	0.90000000	9.04700000	97.32800000
H	-0.77500000	8.51200000	97.17700000
H	-1.32400000	3.70800000	86.46600000
H	-6.99100000	3.20900000	89.46100000
H	5.20600000	5.87200000	91.29500000
H	-4.25300000	14.50300000	100.68200000
H	5.68700000	3.18100000	101.19700000
H	-0.62200000	6.68600000	94.09700000
H	0.54000000	4.46000000	95.89400000
H	1.40400000	6.86900000	88.33000000
O	-0.95000000	5.34100000	90.80400000
H	-0.26400000	5.33900000	91.52100000
H	-1.79100000	5.41200000	91.28100000
P			
C	5.47400000	2.51100000	100.35700000
C	4.42600000	2.71100000	99.28400000
C	4.52700000	3.74500000	98.34600000
C	3.33400000	1.83700000	99.17100000
C	3.59100000	3.89900000	97.32200000
C	2.39100000	1.97400000	98.15700000
C	2.52100000	3.00300000	97.21600000
O	1.57800000	3.07900000	96.23200000
C	4.25900000	6.34100000	91.57900000
N	3.36100000	5.50600000	90.78900000
C	3.76700000	4.64700000	89.85400000
N	4.93800000	4.89100000	89.20500000
N	3.05100000	3.55100000	89.61800000
C	-4.04800000	13.55400000	101.18600000
N	-3.62800000	12.24100000	100.65800000
C	-0.72700000	4.76000000	87.13200000
C	0.26500000	3.89300000	87.92600000
C	-1.84400000	5.37700000	87.98500000
C	-0.36500000	2.63200000	88.53100000
C	-6.30200000	3.93000000	89.91700000
C	-5.45500000	3.80800000	91.16400000
C	-5.72800000	4.55800000	92.31300000
C	-4.37700000	2.91200000	91.21500000
C	-4.97100000	4.42000000	93.47800000
C	-3.61100000	2.75800000	92.36600000
C	-3.90900000	3.51000000	93.50900000
O	-3.13000000	3.30700000	94.61200000
C	0.54800000	4.24600000	91.76600000
O	1.14000000	3.17600000	91.49400000
C	-0.30100000	4.30900000	93.03900000
O	-4.44500000	7.38900000	95.36400000
C	-0.13400000	5.63100000	93.81000000
O	-4.14100000	6.28100000	97.79000000
C	-1.14800000	5.78900000	94.92100000
O	-1.92400000	4.85200000	98.40600000
C	-2.29100000	6.53300000	94.61700000
O	0.61700000	5.32100000	91.06600000
C	-3.29300000	6.69800000	95.58000000
C	-4.59600000	8.02600000	94.09400000
C	-3.15100000	6.11800000	96.85600000
C	-3.84200000	7.28000000	98.77300000
C	-1.99200000	5.36800000	97.14700000
C	-0.80300000	4.02900000	98.72300000
C	-0.99900000	5.19600000	96.17600000
N	1.32800000	7.26100000	88.39400000
C	1.75900000	8.43500000	89.18600000
C	3.30000000	8.44000000	89.20800000
O	3.84100000	7.55000000	88.35200000

O	3.97300000	9.15500000	89.91800000
C	1.12800000	8.40200000	90.58300000
C	1.35100000	9.58100000	91.54300000
C	0.62300000	9.19300000	92.82500000
O	-0.59300000	8.97000000	92.83000000
N	1.39800000	8.99000000	93.92200000
C	0.87000000	8.45000000	95.16100000
C	1.09000000	9.47500000	96.28400000
O	2.18500000	10.01800000	96.41600000
C	1.58500000	7.15100000	95.59300000
S	1.64600000	5.79800000	94.35400000
N	0.04400000	9.69100000	97.11600000
C	0.15500000	10.62600000	98.21600000
C	-1.21100000	10.93100000	98.80900000
O	-2.25100000	10.43200000	98.40100000
O	-1.11600000	11.79500000	99.80700000
H	5.02500000	2.17600000	101.30000000
H	6.21000000	1.75100000	100.06200000
H	5.35700000	4.44600000	98.41100000
H	3.22300000	1.02800000	99.89000000
H	3.69300000	4.70500000	96.59900000
H	1.55500000	1.28700000	98.06600000
H	1.77200000	3.83200000	95.63300000
H	3.66100000	6.78300000	92.37700000
H	4.71000000	7.14800000	90.99800000
H	2.36100000	5.44800000	91.04400000
H	5.12200000	5.86400000	88.97900000
H	5.25300000	4.21300000	88.52600000
H	2.24600000	3.32900000	90.26800000
H	3.10500000	3.09400000	88.71900000
H	-3.75200000	14.33200000	100.47600000
H	-5.12900000	13.63400000	101.36200000
H	-2.05300000	11.99100000	100.18100000
H	-4.09800000	12.02500000	99.77800000
H	-3.89100000	11.49600000	101.30200000
H	-0.16700000	5.56100000	86.62500000
H	-1.45500000	6.00000000	88.79800000
H	-2.46800000	4.60900000	88.45300000
H	-2.50400000	6.00300000	87.37300000
H	0.69500000	4.48100000	88.74200000
H	1.09400000	3.60700000	87.26000000
H	0.37400000	2.03700000	89.07700000
H	-1.14700000	2.89000000	89.25300000
H	-0.81400000	2.00100000	87.75300000
H	-6.95700000	4.80600000	89.96200000
H	-5.68000000	4.02500000	89.01800000
H	-6.55400000	5.26600000	92.30500000
H	-4.13100000	2.32100000	90.33400000
H	-5.20300000	5.01700000	94.35700000
H	-2.77700000	2.06400000	92.40100000
H	-3.31400000	3.99300000	95.27500000
H	0.61900000	11.55900000	97.87800000
H	0.80400000	10.23400000	99.01000000
H	-0.87400000	9.29800000	96.93800000
H	-1.35300000	4.26000000	92.73600000
H	-2.40100000	6.93700000	93.62000000
H	-5.55800000	8.54100000	94.14000000
H	-3.79500000	8.75400000	93.91600000
H	-4.60700000	7.29400000	93.27700000
H	-4.73100000	7.35900000	99.40400000
H	-2.98400000	6.97900000	99.38700000
H	-3.63400000	8.25000000	98.30500000
H	-0.96800000	3.68700000	99.74600000
H	0.14000000	4.59000000	98.67500000
H	-0.73100000	3.16700000	98.05000000
H	-0.13100000	4.58600000	96.38500000
H	0.50900000	7.47100000	87.83000000
H	1.05400000	6.50500000	89.02600000
H	1.46100000	9.35100000	88.65900000
H	0.05400000	8.28100000	90.42700000
H	1.45400000	7.48400000	91.07800000
H	0.89800000	10.49600000	91.14400000
H	2.41700000	9.76300000	91.70000000
H	2.35500000	9.31800000	93.93900000
H	-0.18900000	8.25500000	94.99500000
H	2.63200000	7.37500000	95.82400000
H	1.10700000	6.78400000	96.50600000
H	-1.17100000	4.15400000	86.32900000
H	-6.94100000	3.04800000	89.77600000
H	5.05000000	5.73100000	92.02800000
H	-3.52500000	13.74100000	102.12800000
H	6.02500000	3.43600000	100.55800000
H	-0.28100000	6.44500000	93.10400000
H	-0.10000000	3.44200000	93.67300000
H	3.02500000	7.11100000	87.95500000
O	-1.80700000	6.94500000	91.14600000
H	-1.02600000	6.35500000	91.12300000
H	-1.49700000	7.72300000	91.64400000
